# Induction of Body Weight Loss through RNAi-Knockdown of APOBEC1 Gene Expression in Transgenic Rabbits

**DOI:** 10.1371/journal.pone.0106655

**Published:** 2014-09-12

**Authors:** Geneviève Jolivet, Sandrine Braud, Bruno DaSilva, Bruno Passet, Erwana Harscoët, Céline Viglietta, Thomas Gautier, Laurent Lagrost, Nathalie Daniel-Carlier, Louis-Marie Houdebine, Itzik Harosh

**Affiliations:** 1 INRA UMR1198, Biologie du Développement et Reproduction, Jouy en Josas, France; 2 ObeTherapy Biotechnology, Evry, France; 3 INSERM UMR866, Université de Bourgogne, Dijon, France; 4 INRA UMR1313, Génétique Animale et Biologie Intégrative, Jouy-en-Josas, France; INRA, France

## Abstract

In the search of new strategies to fight against obesity, we targeted a gene pathway involved in energy uptake. We have thus investigated the *APOB* mRNA editing protein (*APOBEC1*) gene pathway that is involved in fat absorption in the intestine. The *APOB* gene encodes two proteins, APOB100 and APOB48, via the editing of a single nucleotide in the *APOB* mRNA by the APOBEC1 enzyme. The APOB48 protein is mandatory for the synthesis of chylomicrons by intestinal cells to transport dietary lipids and cholesterol. We produced transgenic rabbits expressing permanently and ubiquitously a small hairpin RNA targeting the rabbit *APOBEC1* mRNA. These rabbits exhibited a moderately but significantly reduced level of *APOBEC1* gene expression in the intestine, a reduced level of editing of the *APOB* mRNA, a reduced level of synthesis of chylomicrons after a food challenge, a reduced total mass of body lipids and finally presented a sustained lean phenotype without any obvious physiological disorder. Interestingly, no compensatory mechanism opposed to the phenotype. These lean transgenic rabbits were crossed with transgenic rabbits expressing in the intestine the human *APOBEC1* gene. Double transgenic animals did not present any lean phenotype, thus proving that the intestinal expression of the human *APOBEC1* transgene was able to counterbalance the reduction of the rabbit *APOBEC1* gene expression. Thus, a moderate reduction of the APOBEC1 dependent editing induces a lean phenotype at least in the rabbit species. This suggests that the *APOBEC1* gene might be a novel target for obesity treatment.

## Introduction

Obesity is becoming a major problem all over the world spreading like global epidemic with a higher prevalence in the USA [1]. Overweight and obesity are important risk factors for diabetes and cardiovascular disease. Several hundreds of genes are involved in obesity and the estimation is that one quarter of our genome is involved in weight management and energy metabolism [2], [3]. In the search of new targets for obesity, we have investigated the *APOB* mRNA editing protein (*APOBEC1*) gene pathway that is involved in fat absorption in the intestine.

The *APOB* gene encodes two proteins, APOB100 and APOB48, via the editing of a single nucleotide in the mRNA by a specialized enzyme, the *APOB* mRNA editing protein (APOBEC1). This enzyme, a catalytic deaminase expressed in human and rabbit in the intestine but not in the liver, is part of a complex that deaminates a cytidine residue to an uridine one in the intestine *APOB* mRNA (at position 6666 in the human and 6529 in the rabbit) thus generating a STOP codon; it results in the production of the shorter polypeptide designated APOB48 [4] [5] [6]. APOB48 is essential for chylomicron formation, secretion and transport of dietary cholesterol and triglyceride from the intestine [7], [8]. Besides, in the liver, where the editing protein is not expressed, and editing does not occur, the unaltered mRNA gives rise to APOB100 that is an integral part of VLDL and LDL.

With the aim to show that *APOB* mRNA editing is a target mechanism for fighting against obesity, we searched to modulate APOBEC1 enzymatic activity *in vivo* in the rabbit species by modulating *APOBEC1* gene expression through transgenesis. Rabbits have the same lipid metabolism as human [9] as opposed to mice that express *APOBEC1* gene both in the liver and intestine [10], do not have CETP and have higher level of HDL and lower level of LDL, that altogether makes mice a less suitable model to study lipid metabolism than rabbits. Thus, we generated transgenic rabbits by knocking down the endogenous *APOBEC1* gene using RNA interference strategy and expressing permanently a small hairpin RNA (shRNA) targeting specifically the rabbit *APOBEC1* mRNA. We generated also transgenic rabbits expressing the human *APOBEC1* gene, and double transgenic animals by inter-crossing these two models. We observed interesting differences in the phenotypes of these rabbits, especially as regard to their body weight and total lipid content. Finally, our results suggest that APOBEC1 could be considered as a potential target for metabolic disorder treatment.

## Results

### Production of transgenic rabbits

**Figure 1:**
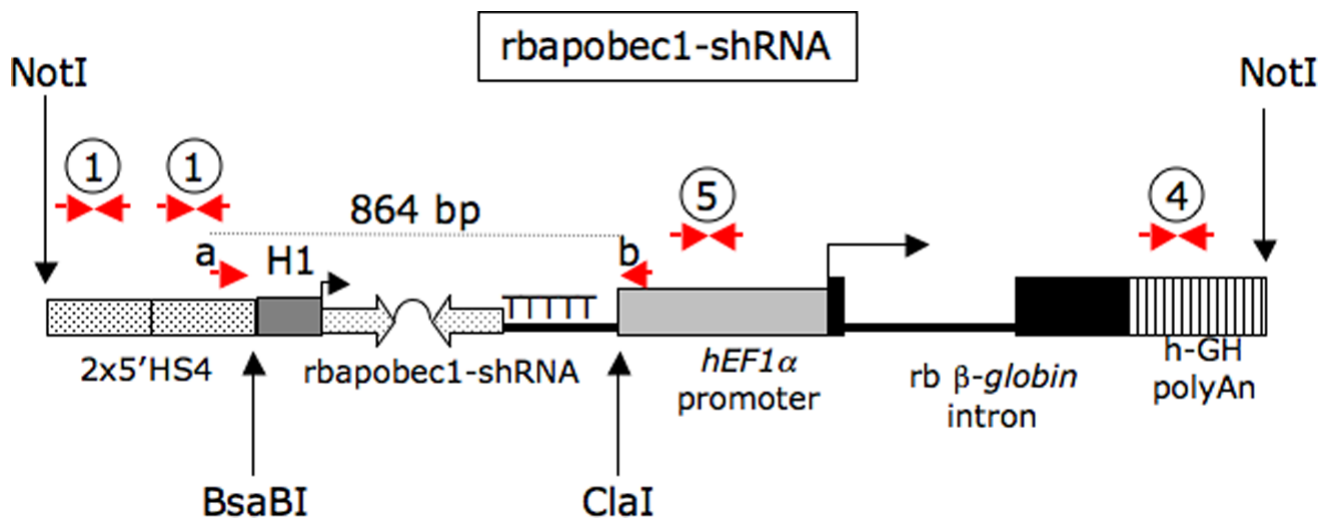
Structure of the rbapobec1-shRNA construct. The rbapobec1-shRNA construct encompassed the H1-rbapobec1-shRNA gene that expressed the shRNA under the activity of the H1 promoter. A gene expression insulator element (two copies of the chicken ß-*GLOBIN* gene fragment 5′HS4) and a transcription unit composed of the *hEF1alpha* – promoter, the rabbit ß-*GLOBIN* second exon and intron, and the human *GH* gene polyadenylation signal were expected to protect the shRNA expression from transcriptional extinction that occurs frequently in transgenesis. Transgenic animals were detected by PCR using sets of primers 1, 4, and 5 (Table S1). Moreover, we checked that after PCR amplification using the a/b set, a 864 bp long fragment with the expected sequence was amplified.

We aimed to produce transgenic animals expressing a shRNA targeting the rabbit *APOBEC1* mRNA in order to knock down the expression of this gene. A construct encompassing a shRNA expressing gene (rbapobec1-shRNA, Figure 1) was therefore introduced by microinjection in the pronuclei of fertilized unicellular rabbit embryos. The sequence of the shRNA targeting the rabbit *APOBEC1* mRNA was chosen among a set of sequences designed by using the OligoWalk tool [11] after assessment of its high efficiency by using an *in vitro* test as previously described [12] (Figure S1).

Twenty-five rabbits were born after microinjection of the rbapobec1-shRNA construct in pronuclei of unicellular rabbit embryos. The screening of newborn rabbits led us to identify 5 (20%) rbapobec1-shRNA transgenic founders. Transgenic lines were successfully established from 3 (shL21, shL23, shL27) of these founders by breeding each one with a wild type animal of the facility. One copy of integrated transgene was integrated in each line. The efficiency of transgenesis and germline transmission was similar to what is currently observed in our rabbit transgenesis facility and led us to suppose that the transgenes were not deleterious for the survival of the rabbits.

### rbapobec1-shRNA transgene expression

**Figure 2:**
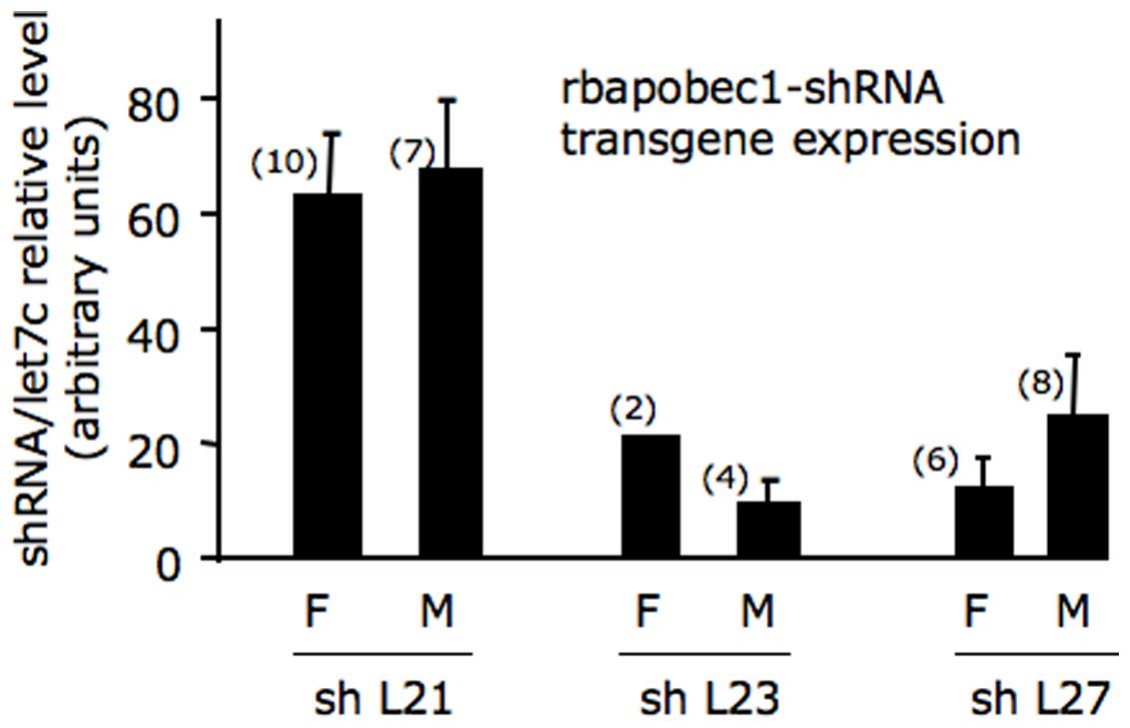
rbapobec1-shRNA transgene expression in rabbit intestine. The amount of shRNA targeting the rabbit *APOBEC1* mRNA was measured in RNAs prepared from duodenum cells as described in “materials and methods” section in 3 rbapobec1-shRNA lines (shL21, shL23 and shL27). Values are given in females (F) and males (M) after normalization to the level of Let7c miRNA determined simultaneously as reference gene in each sample. The number of animals in each group is indicated in brackets. Values are given with the standard error of the mean (sem). All shRNA expressing lines harbored one copy of the rbApobec1-shRNA transgene. Note that in shL21 line, the shRNA transgene expression was the hig hest compared with lines shL23 and shL27.

The rbapobec1-shRNA transgene was expected to produce a shRNA able to knock down the expression of the rabbit *APOBEC1* gene that is known to be specifically expressed in the intestine [13]. The transgene expression was measured in scrapped duodenum cells. Within each line, the expression of the rbapobec1-shRNA transgene was stable over generations and not significantly different in males and females (Figure 2). Note that in shL21 line, the shRNA transgene expression was the highest compared to lines shL23 and shL27. The line shL23 was not further studied.

### Expression of the rabbit *APOBEC1* gene

**Figure 3:**
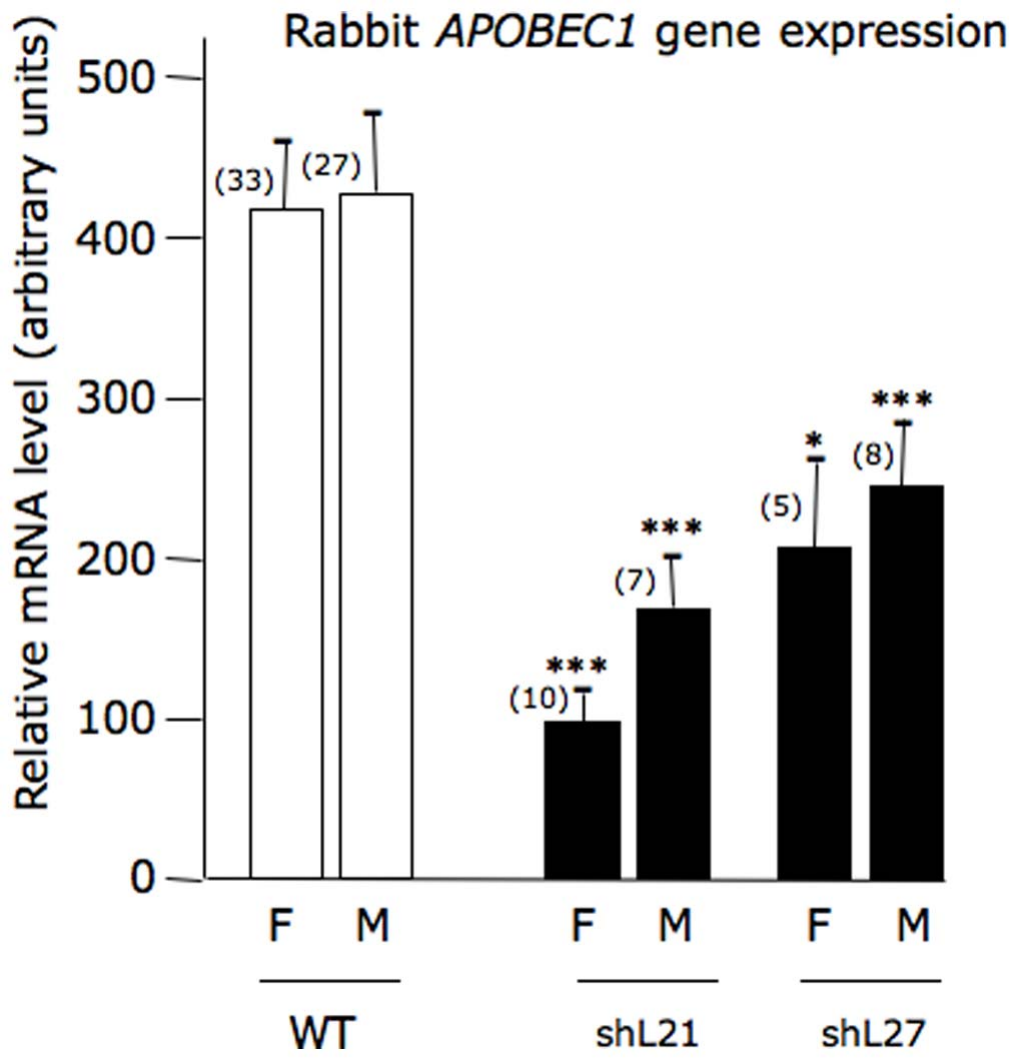
Expression of the rabbit *APOBEC1* gene in wild type and rbapobec1-shRNA transgenic rabbits. The amount of rabbit *APOBEC1* mRNA was measured in RNAs prepared from duodenum cells as described in “materials and methods” section in wild type animals (WT) and in two rbapobec1-shRNA lines (shL21, shL27). Values are given in females (F) and males (M) after normalization to the level of expression of three reference genes (*RPLT9, YHWAZ, HPRT*) determined simultaneously in each sample. The number of animals in each group is indicated in brackets. Values are given with the standard error of the mean (sem). Comparisons were made with control animals of the same sex (*** = p<0.001; * = p<0.05).

As presented in Figure 3, the level of the rabbit *APOBEC1* gene expression was moderately (2 to 3 times) but significantly reduced in both males and females in the rbapobec1-shRNA lines shL21 and shL27. This suggests that the shRNA produced by the rbapobec1-shRNA transgene targeted the rabbit *APOBEC1* gene probably through a RNA interference mechanism.

Unfortunately, no antibody was available to detect by Western blot the rabbit APOBEC1 protein in intestinal cell extracts. Thus we are unable to confirm that the level of rabbit APOBEC1 enzyme was lower in rbapobec1-shRNA transgenic animals than in wild type ones.

### Indirect quantitative estimation of the level of APOB mRNA editing in intestinal cells

In numerous mammals, it has been already reported that the APOBEC1 induced *APOB* mRNA editing introduces a STOP codon in the *APOB* mRNA [14]. In the rabbit species, this phenomenon is responsible for the conversion of a C residue in a U one at the 2177^th^ codon of the rabbit *APOB* mRNA [15]. We have attempted to quantify the level of editing in the various transgenic lines and in wild type animals to test whether the reduction of *APOBEC1* gene expression could modify the *APOB* mRNA editing.

**Figure 4:**
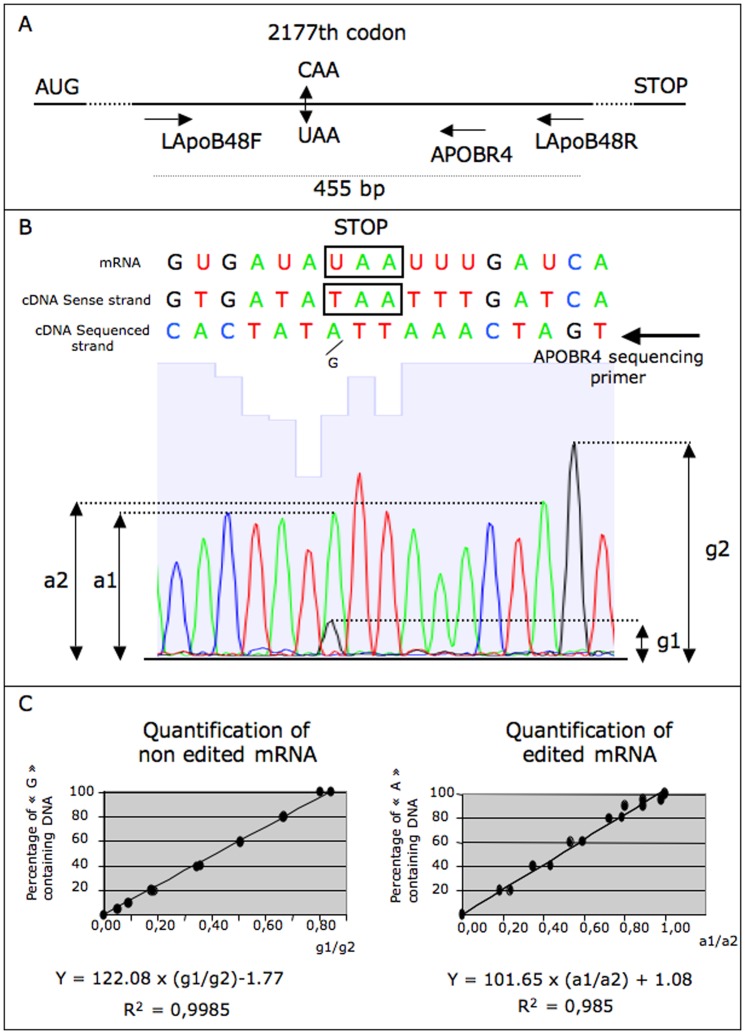
Indirect estimation of the level of “CAA” to “UAA” editing. **A:** schematic representation of the rabbit *APOB* mRNA from the AUG translation initiation codon until the STOP codon. At the 2177^th^ codon, the “C” residue is edited in a “U” residue. Using reverse transcribed RNA as template, the LApob48F/LApoB48R set of primers amplifies a 455 bp long amplicon encompassing the 2177^th^ codon. When using the APOBR4 primer as sequencing primer, the chromatogram shows the antisense sequence. **B:** detail of a characteristic chromatogram showing how the heights of the peaks were measured at the level of the 2177^th^ codon. Here, the “A” residue was the major one (a1), and the “G” the minor one (g1). Consequently, a large majority of DNA strands in this mixture encompassed the edited TAA (STOP) codon at position 2177. (a2) and (g2) are measured as references. **C:** standard equations obtained by plotting the a1/a2 and g1/g2 ratios against the amount of “A” or “G” containing DNA 455 bp fragment in the sequenced sample. Amounts are given as percentage of “A” or “G” containing DNA.

This was achieved by analyzing the chromatograms of the sequence of DNA fragments encompassing the edited nucleotide and produced in each animal by PCR using reverse transcribed intestinal RNAs as template and the LapoB48F/LapoB48R set of primers (Figure 4A and Table S1). Editing was responsible for the modification of the “C” nucleotide in a “T” one at the expected position in the amplified product. We postulated that after amplification, the yield of amplified fragment with a “T” residue was similar to the yield of edited mRNA. We deduced the later from the height of the peaks of each sequence chromatogram (Figures 4B and 4C).

**Figure 5:**
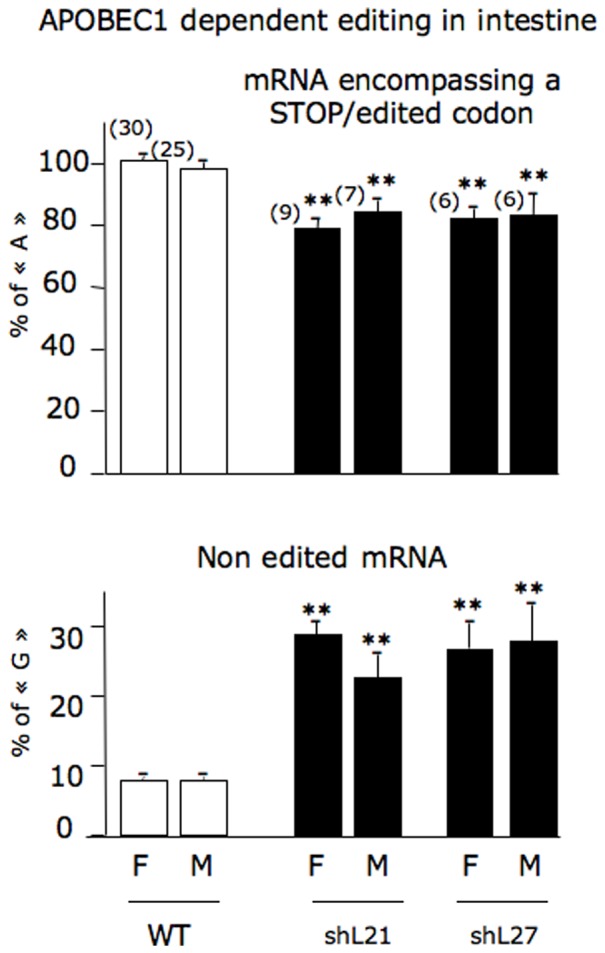
Indirect estimation of editing in wild type and rbapobec1-shRNA transgenic rabbits. APOBEC1 dependent editing was measured in the intestine of wild type and transgenic animals. Values were deduced from sequence chromatograms of a PCR fragment encompassing the edited codon as described in “material and methods” section and in Figure 4. The amount of DNA with a “A” residue was representative of the amount of *APOB* mRNA with a 2177^th^ STOP/edited codon; the amount of DNA with a “G” residue was representative of the amount of full length *APOB* mRNA. The number of studied animals in each group is indicated in brackets. Mean values are given as percentages with the standard error of the mean (sem). Comparisons were made with control animals (** = p<0.001).

Typical chromatograms are presented in Figure S2 showing that in wild type rabbits, editing occurred in the intestine and not in the liver. More than 95% of *APOB* mRNA was edited in the intestine in wild type animals (Figure 5). Interestingly, the level of editing was clearly lower in rbapobec1-shRNA expressing lines (shL21 and shL27, Figure S2 and Figure 5), with a significant reduction of the number of STOP/edited codon (UAA) encompassing *APOB* mRNA and a concomitant significant increase of the number of non-edited *APOB* mRNA. This led us to propose that the alteration of the level of intestinal editing in shRNA expressing animals was consecutive to the probable reduction of the level of APOBEC1 enzyme in this tissue.

### APOB48 amount in plasma

The APOB48 protein is produced by the translation of the *APOBEC1* dependent edited *APOB* mRNA. Since the *APOBEC1* gene expression and the APOBEC1 dependent editing differed in wild type and rbapobec1-shRNA expressing rabbits, it was expected that the plasma level of APOB48 protein also differed in these rabbits.

**Figure 6:**
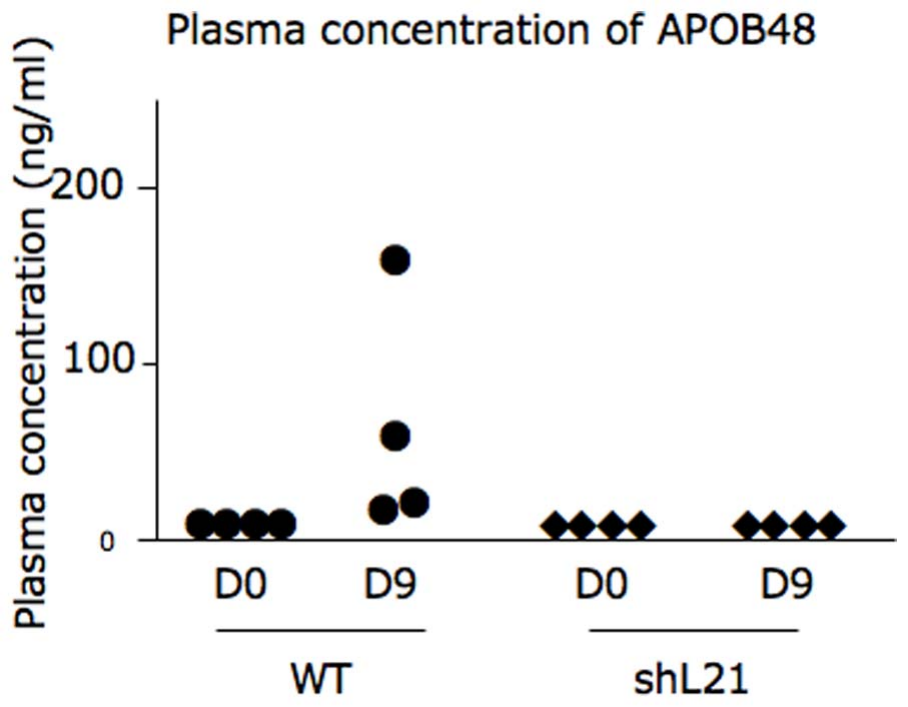
Plasma concentration of APOB48 in rabbits challenged by a high fat/high cholesterol regimen. Plasma concentration of APOB48 was assayed by a specific ELISA kit. Four wild type rabbits and four transgenic rabbits expressing the rbapobec1-shRNA transgene were fed ad libitum with a high fat/high cholesterol regimen for 9 days. Blood samples were collected before the high fat/high cholesterol regimen (D0) and 9 days after the starting of the regimen (D9). Each point indicates the plasma concentration of APOB48 (in ng/ml) in one animal.

No efficient antibody was available to detect the rabbit APOB48 protein by Western blot in intestinal extracts. However, we attempted to assay the concentration of APOB48 in the plasma of rabbits using an ELISA specific for the rabbit APOB48 [16]. Firstly, we assayed APOB48 in all plasma samples collected when animals were sacrificed. Surprisingly, all values were similar to the background level of the ELISA. In Kinoshita's paper, it was reported that the plasma level of APOB48 was enhanced in rabbits fed for at least 8 days with a cholesterol- and triglyceride-enriched regimen. Thus, we decided to feed wild type rabbits and rbapobec1-shRNA expressing rabbits with a soybean oil (8%) and cholesterol enriched (0.2%) regimen [17]. As shown in Figure 6, the plasma level of APOB48 was significantly detected in all wild type animals after feeding for 9 days with the high fat regimen. Besides, the plasma level of APOB48 was not detected in any rbapobec1-shRNA transgenic animal on the four that have been tested. We propose that the undetectable level of plasma APOB48 in plasma samples of most rbapobec1-shRNA transgenic animals was the consequence of the reduction of *APOBEC1* gene expression in the intestine and of the modification of the *APOB* mRNA editing.

### APOBEC1-mediated changes in plasma lipid levels and lipoprotein distribution

**Figure 7:**
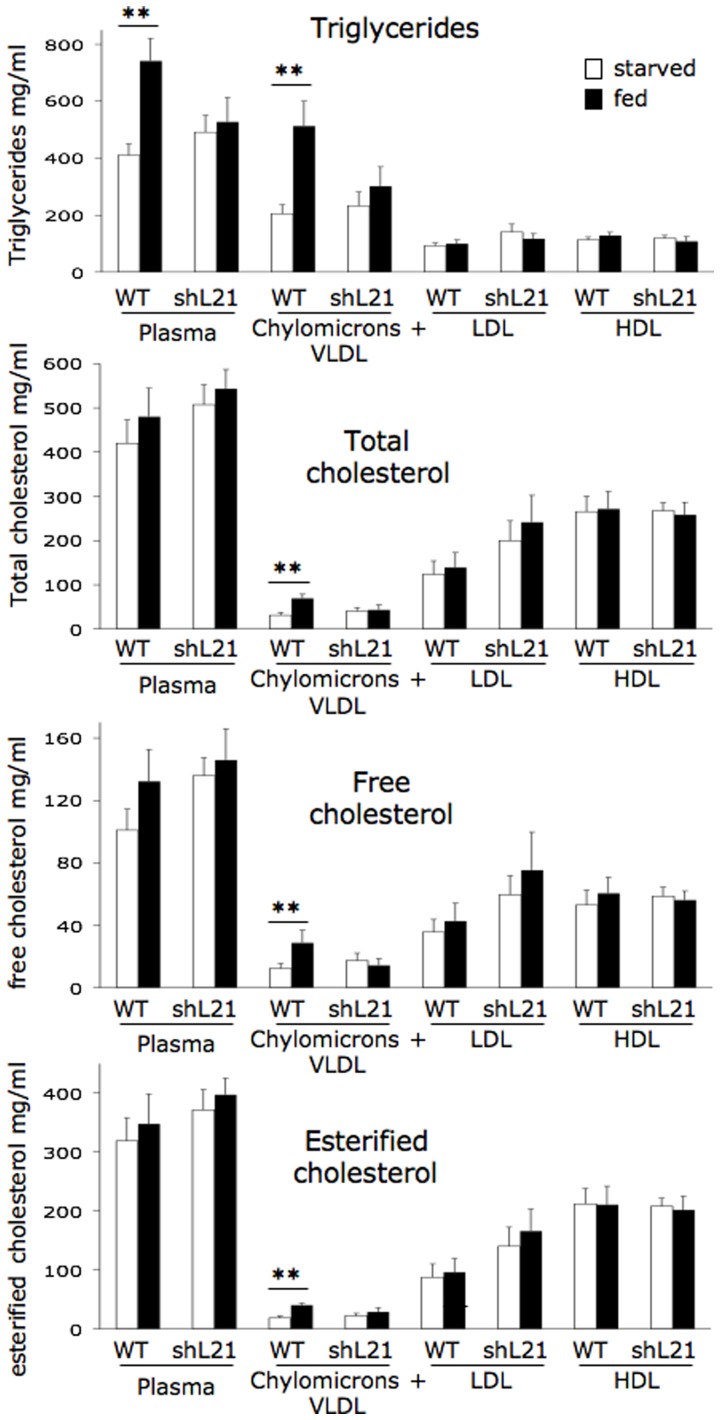
Plasma concentrations of triglycerides and cholesterol (total, free and esterified) in rabbits fed with a normal diet. Triglycerides and cholesterol were assayed in the plasma, and in three lipoproteic compartments separated by ultracentrifugation. Blood samples were collected in rabbits (6 wild-type and 7 transgenic rabbits from line shL21) fed with a normal diet and starved for 20 hours (white bars) and 4 hours after re-feeding with the normal diet (black bars). Values are given in mg/ml, with the standard error of the mean. Comparisons were made between starved and fed animals within each group (** = p<0.001).

We hypothesized that chylomicron formation and secretion were impaired in rbapobec1-shRNA transgenic rabbits as a consequence of the reduction of intestinal *APOB* mRNA editing, leading to modifications of the transport of dietary cholesterol and triglyceride from the intestine. With the aim to assess the extent of this phenomenon, we analyzed the concentration of cholesterol and triglycerides in the various lipoproteic fractions of the plasma. Cholesterol (total, free and esterified) and triglycerides were assayed in rabbits fed with a normal diet. The daily food intake was not different in transgenic and wild type animals. Plasma samples were collected after 20 hours fasting, and 4 hours after re-feeding. As shown in Figure 7, after 20 hours fasting, and in all classes of lipoproteins, the concentration of lipids was not different in wild type and in transgenic rabbits (comparison of white bars in WT and shL21 rabbits in each lipid fraction). Besides, after re-feeding, the expected increase of triglycerides and cholesterol in the chylomicron + VLDL fraction was significantly reduced in rbapobec1-shRNA transgenic animals (line shL21, comparison of starved and fed rabbits in each category). Indeed, after feeding, the levels of cholesterol and triglycerides increased clearly in the chylomicron + VLDL fraction in wild type animals only, and not significantly in transgenic animals. As regard to the other classes of lipoproteins (LDL and VLDL) and after re-feeding, there were no significant differences between wild type and shRNA transgenic animals.

**Figure 8:**
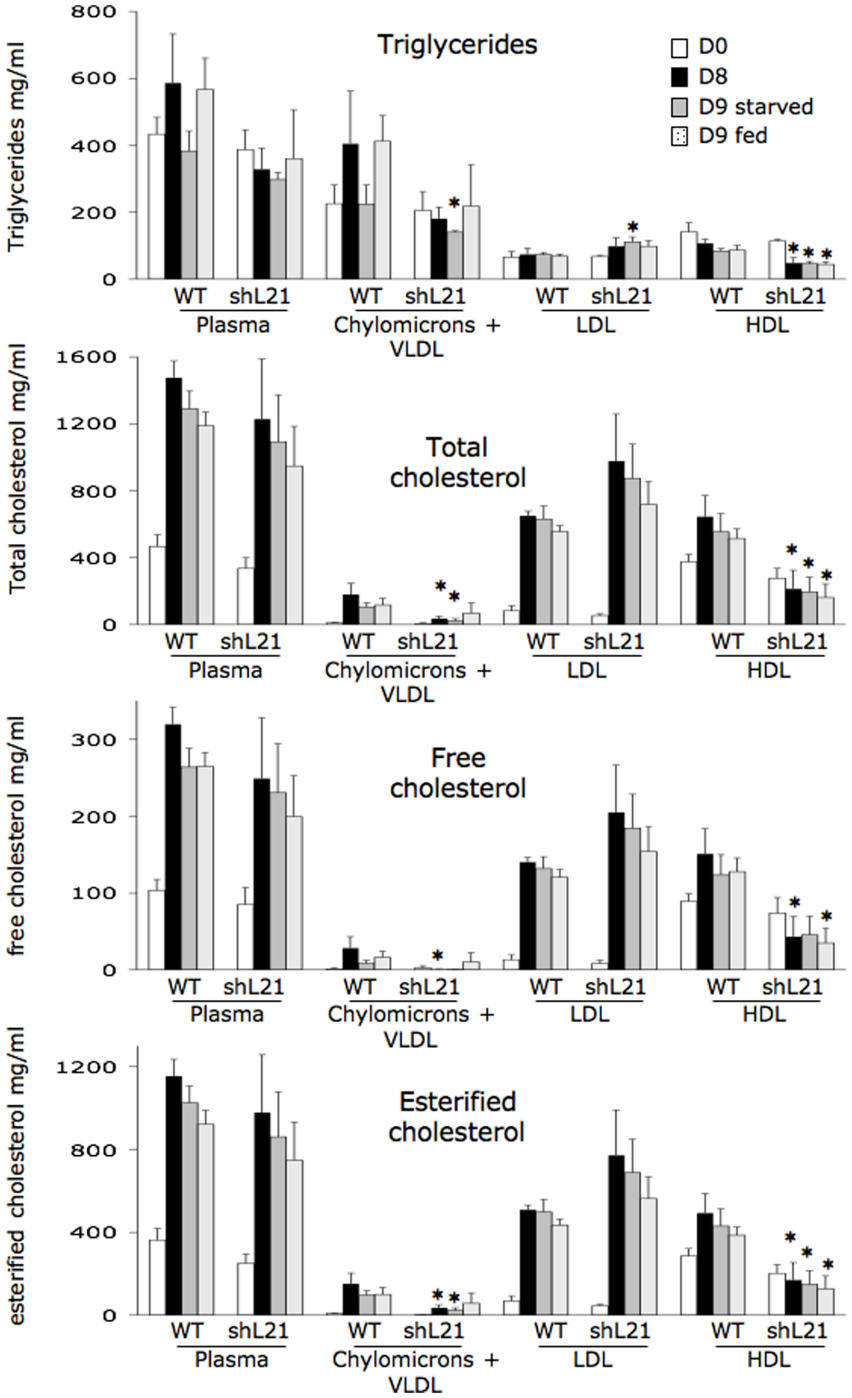
Plasma concentration of triglycerides and cholesterol in rabbits fed with a high fat/high cholesterol regimen. Rabbits (4 wild type, and 3 transgenic rabbits from line shL21) were fed for 8 days with a high fat/high cholesterol diet. Plasma samples were collected before the diet (D0, white bars), after feeding for 8 day with the diet (D8, black bars), after 20 hours starvation (D9 starved, grey bars) and 4 hours after re-feeding with the high fat diet (D9 fed, dotted bars). Triglycerides and cholesterol were assayed as in Figure 7. Values are given in mg/ml, with the standard error of the mean. Comparisons were made between transgenic and wild type animals for each day of the challenge (* = p<0.05).

The ordinary diet of the rabbit is devoid of cholesterol and poor in lipids (around 2% instead of 8% in the high fat diet). To further investigate the lipoprotein distribution in the rbapobec1-shRNA transgenic rabbits and their ability to respond to a high fat diet challenge, animals were fed with a diet enriched with triglycerides and cholesterol. In this experiment, as in the alimentary challenge performed with the normal diet, the daily food intake was not different in transgenic and wild type animals. Triglycerides and cholesterol were assayed in plasma samples collected after 8 days feeding with the enriched diet, after a further 20 hours fasting, and 4 hours after re-feeding with the enriched diet. The pattern of triglycerides concentration in the plasma differed clearly in wild type and transgenic animals (Figure 8). Indeed, the plasma concentration of triglycerides was not enhanced after high fat feeding in transgenic animals as it was in wild type rabbits. More precisely, the chylomicrons + VLDL fraction was not enhanced by the diet challenge in transgenic rabbits. Taken altogether, these data support the hypothesis of an inability of the transgenic animals to produce rapidly large amounts of chylomicrons + VLDL in response to the food supply. The food intake being similar in all animals, this suggests a lower lipid absorption in transgenic animals than in wild type ones.

Interestingly, in the high fat diet animals, and not in the normal diet ones, the concentration of triglycerides and cholesterol in the HDL fraction was obviously reduced in transgenic animals compared to wild type animals. This could be related to the low rate of synthesis of chylomicrons by the intestine in transgenic animals, since chylomicrons and their remnants contribute significantly to the production of HDL.

### Storage of total body lipids

**Figure 9:**
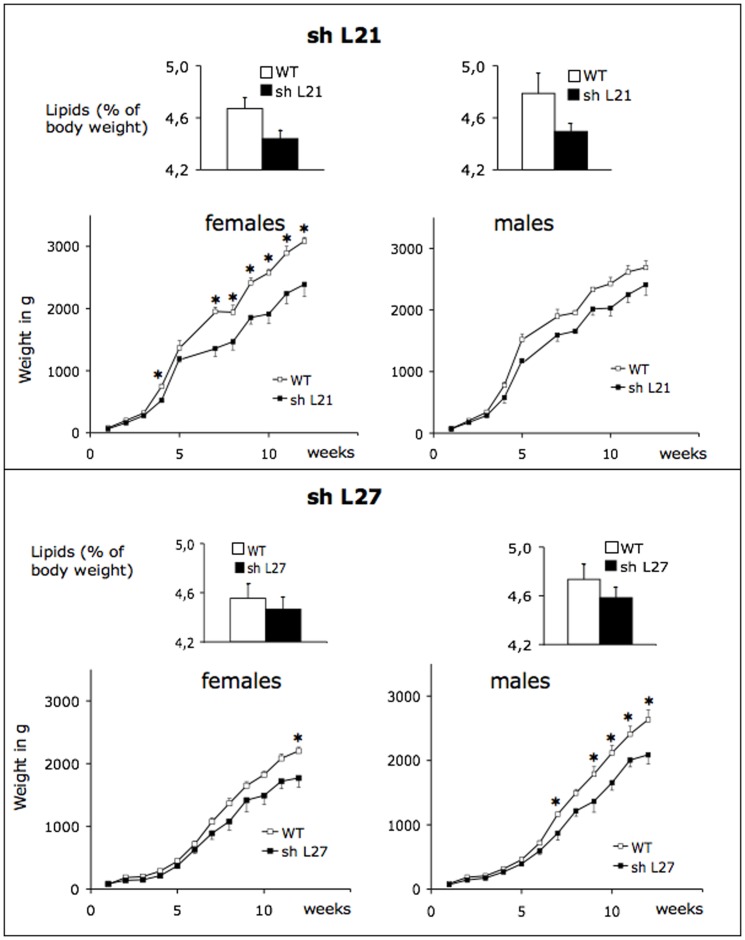
Total content of body lipids and growth curves of wild type and transgenic rabbits from lines shL21 and shL27. The total content of body lipids and growth curves were established on the same rabbits. All rabbits (mothers during pregnancy and lactation and their litters after weaning) were fed with the normal diet. Wild type mothers nourished all newborns (transgenic or wild type ones). The total content of body lipids, expressed as the percentage of the body weight, was measured in transgenic (shL21 and shL27, black bars) and wild type (white bars) rabbits at around 12–16 weeks after birth. Three animals at least were considered for each point. Values are means +/− sem. Note that the percentage was always the lowest in shRNA expressing animals, and the highest in wild type animals. Growth curves were established by weighing weekly each rabbit from 3–5 weeks to 12–18 weeks after birth. Males and females are shown in separate graphs. * = p<0.05 comparison of shRNA expressing animals and wild type ones.

Since the production of chylomicrons was impaired in transgenic rabbit, one could expect that the uptake of lipids from the diet would be reduced leading to a decreased storage of lipids. To assess this hypothesis, the total mass of body fat was estimated using TOBEC analysis at around 12–16 weeks after birth. The total mass of fat was always the lowest in rbapobec1-shRNA transgenic animals (Figure 9), and the highest in wild type animals. We propose that the reduced body mass of lipids in rbapobec1-shRNA transgenic animals was the result of the reduced uptake of diet lipids consecutive from a low production of chylomicrons and low absorption of fatty acids.

### Growth curves in transgenic and wild type rabbits

Our main objective was to study whether modifications of *APOBEC1* gene expression in the intestine induced a lean phenotype in the rabbit species. Thus, all animals were weighed weekly from birth during 12–18 weeks. All transgenic litters were obtained by breeding a transgenic male with a wild type female. Newborns being thus nourished by wild type mothers, this eliminated any possible incidence of the transgenic milk on growth.

At birth, the weight of newborns was not significantly different whatever animals were transgenic or not. However, after three weeks and for the whole length of the experimentation, transgenic rabbits expressing the shRNA targeting the rabbit *APOBEC1* gene (shL21 and shL27) were always the lightest animals (by 10% to 20%) as shown within each litter (Figure 9). Thus, this led us to conclude that the rbapobec1-shRNA transgene expression induced actually a lean phenotype in the rabbit species. The lean phenotype could result from the low production of chylomicrons + VLDL possibly leading to a reduced uptake of diet lipids and a reduced absorption of energy deriving from fatty acids. However, additional experiments should be performed to confirm this hypothesis, and specifically to study whether the energy expenditure was affected in a different manner in transgenic and wild type animals.

### Rescue of the normal phenotype in double transgenic rabbits expressing both the rbapobec1-shRNA and the human *APOBEC1* gene

In order to eliminate the possibility that the lean phenotype was not consecutive to the reduction of rabbit *APOBEC1* gene expression but was due to any other phenomenon induced by the rbapobec1-shRNA transgene, we decided to produce double transgenic rabbits expressing simultaneously the rbapobec1-shRNA transgene and the human *APOBEC1* gene.

**Figure 10:**
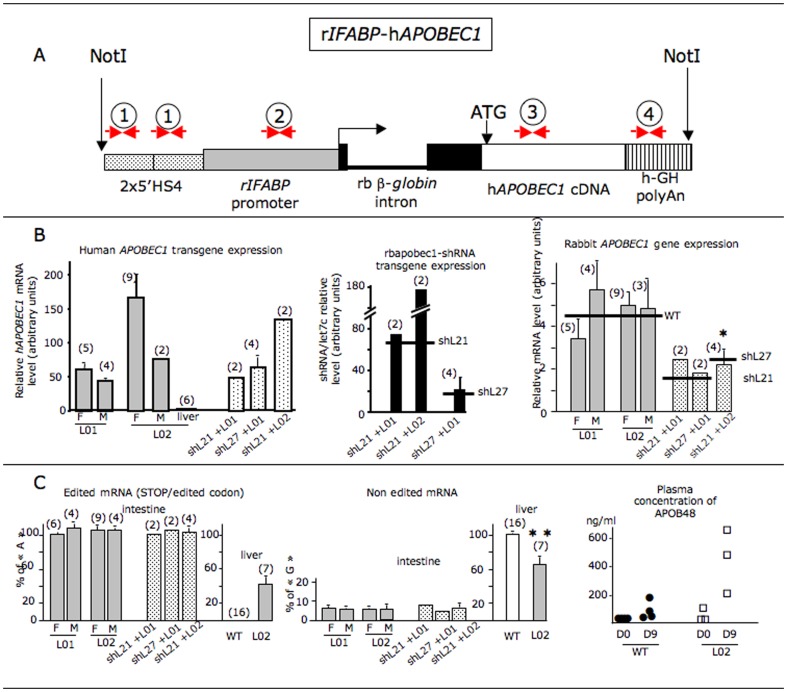
Analysis of rIFABP-hapobec1 transgenic and double transgenic rabbits. **10A: Structure of the recombinant gene to express the human APOBEC1 cDNA in the intestine of transgenic rabbits.** The rIFABP-hAPOBEC1 construct encompassed two copies of the chicken ß-*GLOBIN* gene fragment 5′HS4 (gene expression insulator element, dotted box), the promoter of the rat intestinal fatty acid binding protein gene (r*IFABP*; grey box), the rabbit (rb) ß-*GLOBIN* second intron (black boxes and thick line), the human *APOBEC1* cDNA (white box) produced by PCR amplification from reverse transcribed RNA of HT29 cells (derived from a human colon tumor that have retained the ability to express the *APOBEC1* gene), and the human growth hormone polyadenylation sequences (box with vertical bars). The horizontal black arrow points the position of the transcription start site. ATG =  translation initiation site of *hAPOBEC1* cDNA. Numbers and small horizontal arrows represent the sets of primers. All studied transgenic animals were PCR positive for the sets 1–4. **10 B: gene expression.** The levels of human *APOBEC1* mRNA (left panel) and shRNA (middle panel) were measured in RNAs prepared from duodenum cells in two rIFABP-hapobec1 lines (L01 and L02) and in double transgenic animals (shL21+L01; shL21+L02; shL27+L01). Values are given in females (F) and males (M) after normalization to the level of reference gene expression determined simultaneously in each sample: Let7c miRNA in the case of shRNA, and *RPL19, YHWAZ, HPRT* in the case of human *APOBEC1*. In double transgenic animals, males and females were not distinguished, considering the small number of animals in these groups. The number of animals in each group is indicated in brackets. Values are given with the standard error of the mean (sem). The mean level of shRNA in shL21 and shL27 as presented in Figure 2 is indicated with a horizontal line. The level of expression of the human *APOBEC1* transgene measured in the liver is given in L02. In L01, this level was not significantly detected. The level of rabbit *APOBEC1* mRNA (right panel) was measured in intestinal RNAs as described in Figure 3. The level found in wild type rabbits and in lines shL21 and shL27 is indicated with a horizontal line. **10 C: APOBEC1 dependent editing in intestine and liver and Plasma concentration of APOB48 in rabbits expressing the human APOBEC1 gene.** The estimation of editing was made as described in the legend of Figure 5. Plasma concentration of APOB48 was performed as described in legend of Figure 6 in 3 transgenic rabbits from line L02. Wild type animals are the same than those in Figure 6.

**Figure 11:**
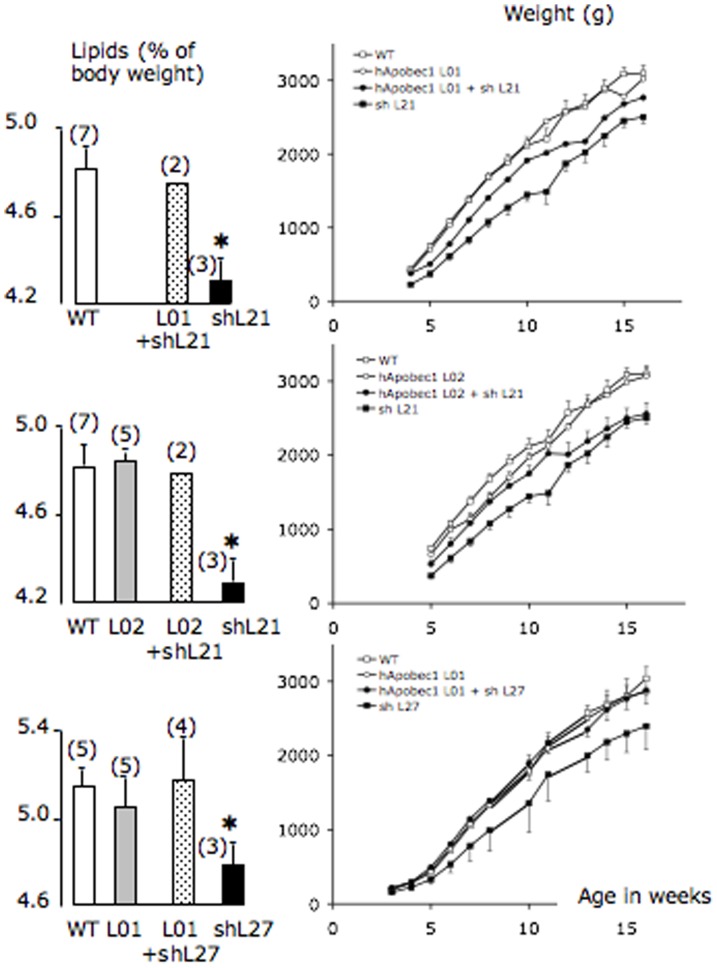
Total mass of body lipids and growth curves in double transgenic rabbits. Double transgenic animals (shL21+L01; shL21+L02; shL27+L01) were produced by breeding rIFABP-hAPOBEC1 (L01 or L02) and rbapobec1-shRNA transgenic lines (shL21 or shL27). In these litters, the total mass of lipids was significantly lower in shL21 or shL27 transgenic animals than in animals from all other groups (* = p<0.05). Numbers in brackets indicate the number of animals in each group. Growth curves were established by weighing weekly each rabbit from 3–5 weeks to 12–18 weeks after birth. Males and females are shown in separate graphs. * = p<0.05 comparison of shRNA expressing animals and wild type ones.

We first produced transgenic rabbits expressing the human *APOBEC1* gene in the intestine through the tissue specific activity of the rat *IFABP* gene promoter [18] added in the construct (Figure 10A). Fifty-four rabbits were born after microinjection of the NotI insert, giving 4 (7.4%) rIFABP-hAPOBEC1 transgenic founders. Transgenic lines were successfully established from 2 (L01 and L02) of these founders, harboring respectively 2 and 6 copies of the transgene. A small number of double transgenic animals expressing both the human *APOBEC1* gene and the shRNA targeting the rabbit *APOBEC1* mRNA were produced by breeding rIFABP-hAPOBEC1 (L01 or L02) and rbapobec1-shRNA transgenic lines (shL21 or shL27). The analysis of transgenic lines L01, L02 and double transgenic rabbits is presented in Figures 10 and 11.

Both transgenic lines L01 and L02 expressed the human *APOBEC1* gene stably over generations, with similar levels in males and females (Figure 10B, left graph). In double transgenic lines, the level of the human *APOBEC1* gene expression was not significantly different from that in lines L01 and L02, which proves that the shRNA produced by the rbapobec1-shRNA transgene did not alter the expression of the human *APOBEC1* transgene. The presence of the human APOBEC1 enzyme was confirmed in the intestine by western blot assay in line L02 (Figure S3) with the expected 27 kD molecular weight. Interestingly, in the transgenic rIFABP-hapobec1 line L02, an unexpected leaking expression of the rIFABP-hapobec1 transgene was detected in the liver but with a 50 times lower level than in the intestine.

As expected, in double transgenic rabbits, the rbapobec1-shRNA was significantly expressed in the intestine (Figure 10B middle graph).

The level of rabbit *APOBEC1* gene expression was similar in transgenic rabbits expressing the human *APOBEC1* gene and in wild type animals (Figure 10B, right graph). Besides, it was lower in double transgenic rabbits, as we had previously observed in rabbits from lines shL21 and shL27. Thus, as we already suggested, the shRNA targeted the expression of the intestinal rabbit *APOBEC1* gene probably through a RNA interference mechanism, without altering that of the human *APOBEC1* gene.

In transgenic animals expressing the human *APOBEC1* gene, the level of editing was at around 95% of the maximum, as it was previously determined in wild type animals (Figure 10C). This was surprising since we were expecting for an increase consecutive to the additional human APOBEC1 enzyme. Though, the human APOBEC1 enzyme was actually efficient in *APOB* mRNA editing in the rabbit as the rabbit APOBEC1 enzyme. Indeed, editing was observed in the liver of some transgenic rIFABP-hapobec1 animals (Figure 10C, line L02, middle panel) harboring a leaking expression of the human *APOBEC1* transgene in the liver, when editing is never observed in liver in wild type rabbits. Thus, the lack of any modification in *APOB* mRNA editing in transgenic animals over-expressing the APOBEC1 enzyme was not due to the inefficacy of the enzyme but probably the consequence of the saturation of the mechanism of editing.

In double transgenic animals, the level of editing was similar to that of wild type animals, despite the reduced expression of the rabbit *APOBEC1* gene in the intestine. This proves that the human APOBEC1 enzyme expressed in the intestine by the transgene was able to counterbalance the default of rabbit APOBEC1 enzyme due to the shRNA targeting the rabbit *APOBEC1* mRNA.

Interestingly, the plasma level of APOB48 was highly enhanced in the human *APOBEC1* transgenic rabbits L02 by the high fat/high cholesterol diet challenge (Figure 10C, right graph). Since editing was not modified in the intestine of these animals, it is likely that the high plasma concentration of APOB48 originated from the liver, where a significant editing of the *APOB* mRNA was measured consecutively to the leaking expression of the human *APOBEC1* transgene.

The plasma lipid levels and lipoprotein distributions were assayed in human *APOBEC1* transgenic rabbits (L02, Figure S4) submitted to the high fat/high cholesterol diet and starvation/feeding challenge. Surprisingly, the concentrations of triglycerides in the plasma and also in the chylomicrons + VLDL fraction were not enhanced by the diet, by opposition to what we were expecting for in these rabbits characterized by a high level of circulating APOB48. Clearly, in these animals, the high circulating APOB48 did not contribute to a high synthesis of chylomicrons. Other differences were further detected throughout the starvation/feeding challenge. These could be consecutive to the leaking expression of the human *APOBEC1* gene in the liver, which induced the liver editing of the *APOB* mRNA and thus the reduction of the hepatic synthesis of APOB100 protein.

The total mass of body lipids and growth curves were determined from a series of litters including newborns of each genotype (wild type, rbapobec1-shRNA, rIFABP-APOBEC1, and double transgenic animals, Figure 11). The transgenic animals expressing the human *APOBEC1* gene gained weight and possessed a total lipid mass as the wild type animals. This was not surprising since in these transgenic animals, the APOB mRNA editing and the production of chylomicrons were similar to those determined in wild type rabbits. A small number of animals of rIFABP-hapobec1 transgenic lines L01 and L02 were weighed for a longer time (Figure S5), in order to detect possible long-term modifications consecutive to limited but sustained modifications of the level of editing that we might have not been able to detect earlier. However, the weight of transgenic animals was not different from that of wild type ones, showing that even in older animals, the long term-expression of human APOBEC1 enzyme induced no significant over-weight gain.

More interestingly, in spite of the small number of animals of each genotype in the litters, the double transgenic animals were clearly heavier than the shRNA expressing animals and their total mass of body lipids was similar to that of wild type animals. This shows once more that the presence of the human APOBEC1 enzyme was able to counterbalance the effect of the shRNA targeting the rabbit *APOBEC1* gene. Taken altogether, our results suggest strongly that the lean phenotype observed in rbapobec1-shRNA transgenic rabbit was the consequence of the reduced level of *APOBEC1* gene expression.

## Discussion

A great number of genes are devoted to the storage of energy, and it is reasonable to propose that evolution has selected organisms able to survive in scarce conditions thanks to efficient mechanisms of energy storage. Limiting energy uptake and storage is probably a valuable strategy to fight against obesity. Thus, our approach consisted of looking for critical genes in people with lean phenotype. If a monogenic slimness disease resulting from a deficiency of fat absorption can be found, the implicated gene likely plays a critical role in the disease and is a potential target for new anti-obesity drugs. When this gene is not compensated by other mechanisms, it is therefore a powerful target for obesity treatment.

Three human genetic diseases have been described with very similar lean phenotypes: abetalipoproteinemia, hypobetalipoproteinemia, and chylomicron retention disease also known as Anderson's disease [19]. The genes involved in the first two diseases, abetalipoproteinemia and hypobetalipoproteinemia, have now been identified, but it is not yet the case in the Anderson's disease [20], [21]. All three diseases are characterized by a severe reduction or total absence of APOB48 protein in intestinal cells and plasma and of chylomicrons production. This led us to investigate further the possibility of fighting against obesity through regulating APOB48 production. APOB48 resulting exclusively from the translation of the APOBEC1 dependent edited *APOB* mRNA, we decided to target the expression of the *APOBEC1* gene.

The phenotype of mice harboring a complete invalidation of the *APOBEC1* gene has been already reported by a series of laboratories [22]–[25]. As expected, the editing of the *APOB* mRNA was suppressed, and no APOB48 was produced in these mice. It was observed that intestinal fat absorption was less efficient in *APOBEC1* ^−/−^ mice containing only APOB100 than in wild type mice but it was not totally abolished. Probably, APOB100 could replace to some extent APOB48 in chylomicron formation and finally the plasma lipoprotein cholesterol and triglycerides profiles were not different in knock out and wild type mice [7] [8]. In contrast, in the human, APOB100 is not able to form chylomicrons and carry lipids from intestine to liver [26]. Clearly, the metabolism of lipids differs between species, and thus we decided to target the *APOBEC1* gene in another species than the mouse and closer to the human as regard to the metabolism of lipids, in order to investigate whether the *APOBEC1* dependent editing could be a valuable target for fighting against obesity through modulating the lipid uptake.

RNA interference is a natural cellular process mediated by small double strand RNA that induces knockdown of gene expression through mRNA targeting. Here, we produced transgenic rabbits expressing permanently a small interfering RNA (siRNA) targeting the rabbit intestinal *APOBEC1* gene. This was achieved through the introduction in the rabbit genome of a DNA construct expressing a small hairpin RNA by using a strategy that we had followed in a previous study [12]. This strategy had the advantage to provoke the sustained production of the siRNA, and a moderate but significant and permanent decrease of the rabbit *APOBEC1* gene expression. Our objective was to observe long term and prolonged effects of the gene knockdown that is totally different to what can be observed after a total invalidation of the gene. To validate our findings, we produced transgenic rabbits expressing the human *APOBEC1* gene in the intestine, with the aim to rescue the knockdown induced by the RNA interference mechanism.

**Figure 12:**
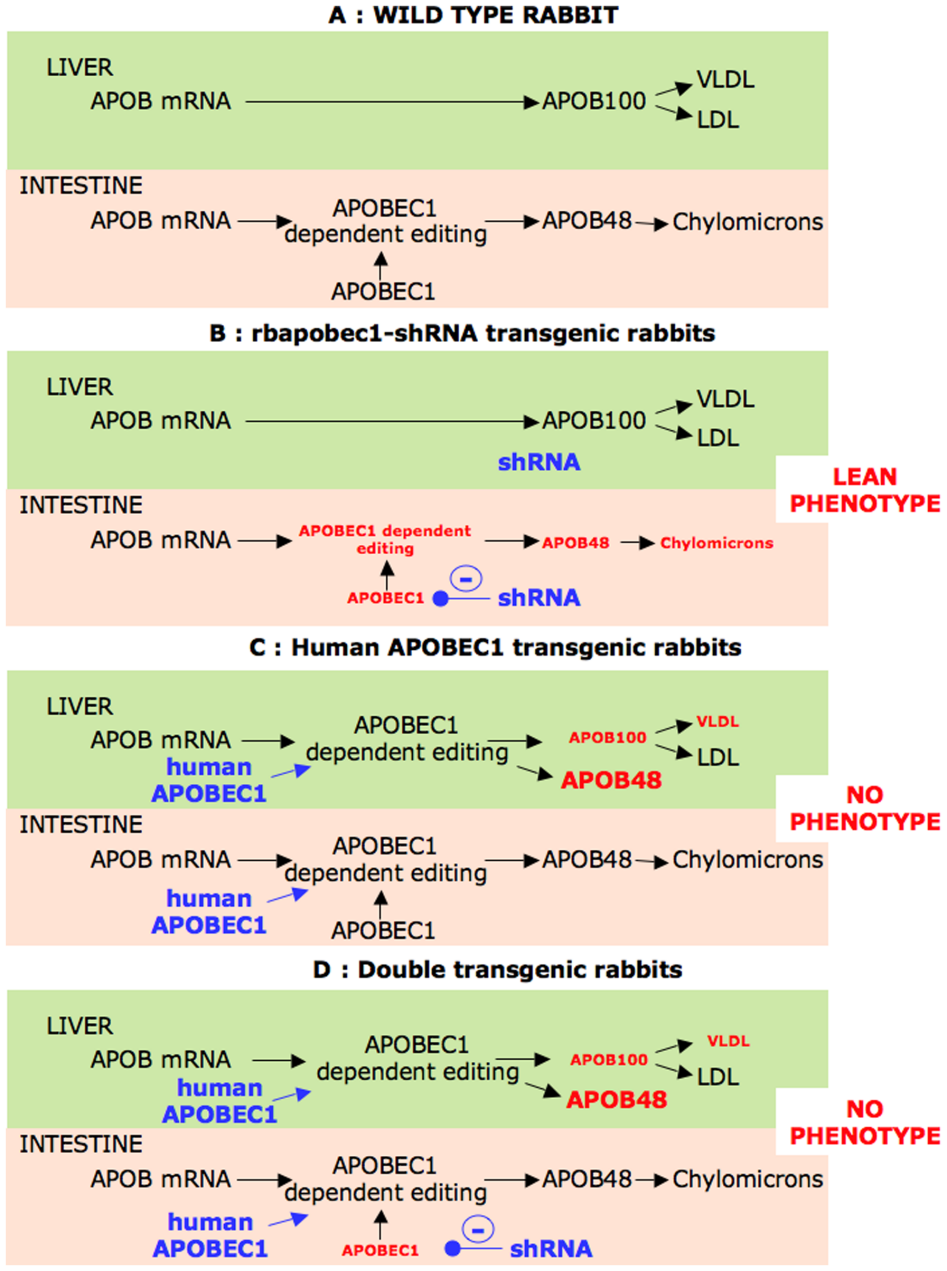
Intestinal and liver regulation of APOB48, APOB100, and chylomicron production. Four schematic representations are given, to simulate the regulation of *APOB* mRNA editing and the consequence upon the phenotype in wild type and transgenic rabbits. The models depict the situation after a diet challenge with normal of high fat/high cholesterol diet. Blue characters are used for the transgene expression (shRNA targeting the rabbit *APOBEC1* mRNA, and human *APOBEC1* gene); red characters indicate the measured parameters with significant modifications; the size of the letters is related to the level of production.

The Figure 12 presents a model that could explain how targeting the *APOBEC1* gene induces a lean phenotype in the rabbit species. In wild type rabbits (Figure 12 A), the *APOBEC1* gene expressed in the intestine only is responsible for the editing of the *APOB* mRNA. The APOB48 protein thus produced exclusively by the intestine is processed to synthesize chylomicrons that are responsible for the lipid uptake from the diet. In the liver, the APOB mRNA is translated in the APOB100 protein that is involved in the synthesis of VLDL and LDL. In transgenic rabbits expressing the rbapobec1-shRNA (Figure 12B), the level of *APOBEC1* gene expression and the level of editing are significantly reduced in the intestine. The ability to synthesize chylomicrons in response to a diet challenge is reduced. The productions of LDL and VLDL are not significantly modified; but the production of HDL is modified since HDL is processed from chylomicrons and remnants that are reduced. The lean phenotype is thus consecutive to the reduced lipids uptake by the enterocytes through the low synthesis of chylomicrons after each food challenge.

The absence of obese phenotype in transgenic rabbits expressing the human APOBEC1 enzyme (Figure 12C) in the intestine is amazing. Indeed, we were expecting for a long-term gain of weight in these animals since the human *APOBEC1* transgene should have enhanced the level of editing of the *APOB* mRNA. It has been already published that in the rabbit species, around 90% of the *APOB* mRNA was edited in the intestine [14]. Accordingly, our results have shown that more than 95% of *APOB* mRNA were edited. However, in the present study, the expression of the human *APOBEC1* transgene was not able to enhance the level of editing, and consequently, animals did not elicit any obese phenotype. One explanation is that the editing was already at its maximum in wild type animals, and that the over-expression of APOBEC1 was inefficient. It has been reported that the editing results from the activity of the APOB mRNA editing complex (the editosome), a multicomponent protein complex with enzymatic and regulatory activities [27]. Thus, we suggest that the editing was limited by the availability or activity of other components of the editing complex.

More surprisingly, in transgenic rabbits expressing the human *APOBEC1* gene, the plasma level of triglycerides in the chylomicrons + VLDL fraction was not enhanced after a fat-rich diet for 8 days (Figure S4) as it was in wild type rabbits. Yet, we were expecting for an enhanced production of chylomicrons, since the plasma level of APOB48 was clearly enhanced in these animals (Figure 10C). Notably, the excess of APOB48 protein was produced by the liver, in response to the leaking expression of the human *APOBEC1* transgene. Thus, it suggests that the APOB48 protein originating from the liver was not processed into chylomicrons, and that only the APOB48 produced by the intestinal cell can form chylomicrons. Finally, this study shows that in spite of a significant high expression of the human *APOBEC1* gene in the intestine, no obese phenotype can be observed in the rabbit species.

In double transgenic rabbits (Figure 12D), the expression of the human *APOBEC1* gene in the intestine counterbalances the shRNA induced knockdown of the rabbit *APOBEC1* gene expression. Consequently, the level of editing is similar to that in wild type animals, as the level of synthesized chylomicrons. The rescue of the normal phenotype in the double transgenic rabbits is a solid argument to demonstrate the relation between *APOBEC1* gene expression and the lean phenotype.

In conclusion, this study presents for the first time evidences that targeting the *APOBEC1* gene is a valuable strategy to induce a lean phenotype in the rabbit. Importantly, it has to be further confirmed that the lean phenotype is the consequence of a moderate modification of the lipid uptake through chylomicrons. Remarkably, animals did not suffer from any disease, and their breeding capacity was not apparently affected for more than two years that is a long experimental duration in this species. However, a series of additional experiments should be performed to investigate the impact of targeting the APOBEC1 gene expression on health, by specifically studying the impact of alterations of intestinal absorption of lipids and other nutrients. Finally, the success of this strategy lies probably in the fact that it concerns a gene pathway without compensatory mechanisms that affects the lipids uptake of the diet. Moreover, it suggests that looking for new genes associated to lean phenotype is probably a valuable tool to highlight novel targets for obesity treatment.

## Materials and Methods

### Animals

Californian rabbits (GD24 strain) were bred at the UCEA rabbit facility (Unité Commune d'Expérimentation Animale, Jouy-en-Josas, France). All experiments were performed with the approval of the local committee for animal experimentation (COMité d'ETHique appliqué à l'Expérimentation Animale (COMETHEA), Jouy-en-Josas, accreditation number 12/017). All researchers working directly with the animals possessed an animal experimentation license delivered by the French veterinary services.

All rabbits were weighed each week from birth until week 12–18, few before puberty that occurs at around 20–24 weeks in this species. Breeders were nourished with a normal diet. Since the growth rate depends on the number of newborns in each litter, care was taken to compare rabbits issued from litters encompassing approximately the same number of newborns. Animals were currently weaned at around 7 weeks. After weaning, animals were fed with the normal diet except otherwise mentioned.

At around 18 weeks after birth, animals were starved for 24 hours, blood samples were collected on EDTA to prevent for coagulation, and food was immediately provided. Four hours after feeding, animals were sacrificed; blood and tissue samples were collected. Blood samples were centrifuged (10 minutes, 3000 g), then plasma and tissues were frozen at −80°C until used.

### Construction of recombinant genes

The rbapobec1-shRNA-producing gene (Figure 1) encompassed two inverted repeats of the rbapobec1-shRNA and a stretch of five T residues as gene terminator. The shRNA transcription unit was constructed from synthetic DNA fragments (Eurofins, Ebersberg, Germany). This H1-rbapobec1-shRNA gene was then inserted into the pM10 vector [12] at the enzymatic BsaBI-ClaI restriction sites as presented in Figure 1. The final construct used for microinjection encompassed the DNA fragment included between the two NotI restriction sites.

The human *hAPOBEC1* construct (rIFABP-*hAPOBEC1*, Figure 10) encompassed the human *APOBEC1* cDNA from 3 nt upstream of the site of initiation of translation (ATG) to 3 nt downstream the STOP codon linked to the rabbit (rb) ß*-globin* second intron. Transcription was driven by the promoter of the rat intestinal fatty acid binding protein gene (r*IFABP*) spanning from nucleotides −1150 to +51 as regard to the *IFABP* gene transcription start site. A tandem of the chicken ß-*globin* gene fragment 5′HS4 was added as insulator. The termination signal was brought by the terminator from the human growth hormone gene (h-GH polyAn).

The sequences of all plasmids encompassing these constructs are available upon request.

### Generation of transgenic rabbits

The inserts to be used for microinjections were released from plasmids by NotI digestion, separated on 1% agarose gel in 1x TBE, purified using the Qiaquick gel extraction kit (Qiagen, Courtaboeuf, France) and then EluTipD filtration (Schleicher & Schuell, Mantes la Ville, France). The resulting DNA preparations were microinjected into Californian rabbit embryo pronuclei at a concentration of 2 ng/µl. The transgenic rabbits were identified using PCR performed on ear clip DNA extracts. Four sets of primers were designed to cover the integrity of each integrated construct: sets 1, 2, 3 and 4 for the *hAPOBEC1* expressing construct, and sets 1, 4, 5 and a/b for the shRNA expressing construct (Figures 1 and 10, table S1). Sets 1, 2, 3, 4, and 5 were used in real time PCR with the fast SYBR Green master mix (Applied Biosystems). In parallel, a set of primers (cas1, cas2, table S1) amplifying a non-coding region upstream of a control gene (the rabbit ß-*CASEIN* gene) was used as control amplification. All sets of primers were designed by the Primer Express software (Applied Biosystem) and all amplicons were 100 base-pairs long.

The number of copies of integrated gene was deduced from real time PCR amplifications by the 2^(Δ (ΔCt))^ method. The sets of primers 1, 2, 3, 4 and 1, 4, 5 were used as transgene specific probes for the rIFABP-hapobec1 and rbapobec1-shRNA constructs respectively. We used the set of primers cas1/cas2 located on the rabbit ß-*CASEIN* gene as reporter probe to normalize to a two copies endogenous gene. A reference rabbit genome was produced by mixing one copy of transgene per copy of genome and used as standard. The number of copies of integrated transgene was similar for sets 1, 2, 3, 4 in the case of the rIFABP-hapobec1 transgene and for sets 1, 4, 5 in the case of the rbapobec1-shRNA transgene, thus suggesting that transgenes were intact.

### shRNA assay

The concentration of shRNA produced by the shRNA constructs in transgenic rabbit tissues was estimated by RT-qPCR [28] [12]. Briefly, 5 µg of total RNA, prepared as previously described [29], were polyadenylated according to Ambion's protocol (PolyA Polymerase, Ambion, Applied Biosystems, France). The polyadenylated RNAs were reverse transcribed (High Capacity cDNA Archive kit, Applied Biosystems) using as reverse primer a polyT adapter encompassing a series of twelve “T” residues and a universal primer (Table S1). Quantification was achieved by SYBR Green quantitative PCR (Applied Biosystems) using a set of primers composed of the universal primer corresponding to the 5′ end of the polyT-adapter and a primer specific to the shRNA sequence, resulting in the amplification of a 65 bp long fragment.

The concentration of shRNA in tissue samples was estimated after normalization by the concentration of Let7c miRNA determined by the same method in each sample. It was thus given by the formula 2^(CtLet7c-CtsiRNA)^. A set of samples was chosen as calibrators and was assayed in all compared runs. Care was taken to consider Ct values within the linear amplification zone.

### Quantification of human and rabbit *APOBEC1* gene expression

Total RNAs were extracted from tissues as previously described [29]. Reverse Transcription (RT) was performed on 1 µg of total RNA using the High Capacity cDNA Archive kit (Applied Biosystems) and the random primer mix included in the kit.

Quantification was achieved using SYBR Green quantitative PCR (fast SYBR Green master mix, Applied Biosystems) with dilutions of the RT reactions and sets of primers designed by the Primer Express software (Applied Biosystem). Whenever possible, primers were chosen on separate exons in order to avoid contaminant DNA amplification, and all amplicons were 100 base-pairs long (Table S1). The sequence of primers was chosen in order to avoid cross reactivity between human and rabbit *APOBEC1* gene measurements. Moreover, for all samples, a RT minus reaction was performed with all RT components except the reverse transcriptase enzyme, and assayed as a complete RT reaction to ensure that no amplification was due to contaminant DNA.

Three normalizing genes (*RPL19, YHWAZ, HPRT*) were tested on all samples for their stable expression in the studied tissues. The GeNorm program included in Biogazelle QBasePlus software (Biogazelle NV, Ghent, Belgium) was used to analyze the data. In order to correct for inter-run fluctuations, a set of samples was chosen as calibrators and was assayed in all compared runs. Care was taken to consider Ct values within the linear amplification zone. Gene expression was considered as significant when Ct values obtained using 2–5 ng of cDNA in each q-PCR reaction were lower than 34, and when one single DNA fragment with the expected size was amplified as template in each q-PCR reaction.

### Characterization of the intestinal human APOBEC1 protein

Human APOBEC1 was characterized in intestinal protein extracts by Western blotting. Scrapped intestinal mucosa cells were homogenized with a Dounce homogenizer in RIPA buffer (50 mM Tris-HCl, pH 7.4; 1% IGEPAL; 0.5% Na-deoxycholate; 0.1% SDS; 150 mM NaCl; 2 mM EDTA; 50 mM NaF; 0.2 mM sodium orthovanadate) with protease inhibitors (Complete Protease Inhibitor Cocktail, Roche; 1 mM PMSF; 1 mM Benzamidine) extemporaneously added. After incubation on ice for 30 minutes and centrifugation at 10 000 g for 10 minutes at 4°C, the supernatant was collected and frozen in aliquots at −80°C. Protein concentration was determined by Bradford assay (BioRad, France) using BSA as standard.

Proteins were separated on a 16% acrylamide gel electrophoresis then transferred on Hybond-P membrane. The human APOBEC1 enzyme was detected after incubation with a rabbit anti-APOBEC1 antibody (Sigma, SAB2100132), a goat anti-rabbit IgG peroxidase conjugate (Sigma, A-0545) and the immunofluorescence ECL 2 detection kit (Pierce).

### Indirect quantification of the proportion of edited ApoB mRNA

Reverse transcribed (RT) RNAs were obtained as for the measurement of rb*APOBEC1* gene expression. A 455 bp long DNA fragment was amplified from RT RNAs using a set of primers specific of the rabbit *APOB* cDNA (LApoB48F/LApoB48R, Table S1). The *APOBEC1* edited 2177^th^ codon of the *APOB* cDNA was included in this amplified DNA fragment (Figure 4A). Editing was responsible for the modification of the “CAA” codon in a “UAA” one (a TAA codon in the amplified product). The amplified fragment was purified by MSB Spin PCRapace (Stratec, Eurobio, France), then sequenced (Eurofins, MWG) using the APOBR4 oligonucleotide as sequencing primer. Using this primer, the antisense strand was sequenced. We measured the yield of 455 bp fragments with a “T” residue in the mixture by analyzing the chromatogram of the sequence of each amplified 455 bp fragment. As shown in Figure 4B, the height of the “G” (g1) and “A” (a1) peaks in the chromatogram was compared to that of the “G” (g2) and “A” (a2) peaks chosen in the vicinity to normalize for sequencing efficiency (in the antisense sequenced strand, “G” and “A” sequenced residues corresponded to the “C” and “T” residue of the edited 2177^th^ codon). A standard curve was performed by sequencing a definite amount of the 455 bp DNA fragment containing a mixture of varying proportions of the two types of DNA strands elsewhere purified, which sequence encompassed the “C” or “T” residue at the 2177th codon (Figure 4C). Two linear equations were deduced by plotting the g1/g2 or a1/a2 ratio against the amount of “C” or “T” encompassing DNA in the mixture. These equations were then used to determine the percentage of “C” or “T” encompassing DNA in each RT RNA mixture produced from the various studied samples. Thus, we indirectly measured the yield of edited mRNA.

### Plasma APOB48 levels

Plasma APOB48 levels were assayed using an ELISA [16] as indicated by the manufacturer (Shibayagi, X-Celtis GmBH, Germany). To enhance the level of APOB48 in the plasma, rabbits were fed ad libitum for 9 days with a high fat/high cholesterol regimen containing 0,2% cholesterol and 8% soybean oil [17].

### Plasma cholesterol and lipid levels

The major classes of lipoproteins were isolated from plasma samples by sequential ultracentrifugation ensuring the separation of chylomicrons + VLDL, LDL and HDL [30]. Cholesterol (total, esterified, and free) and triglycerides were further determined in each fraction using commercially available enzymatic kits.

### Total body content of lipids

The total content of lipids was deduced from the measure of total body electrical conductivity (TOBEC) as previously described [31] with modifications brought by L Lamothe and C Bannelier for using an EM-SCAN SA-3000-type chamber. Animals were not anesthetized during measurements. Measurements were made at around 12-16 weeks after birth, and rabbits were immediately weighed. The content of lipids was deduced from the E-value given by the TOBEC and the weight of the rabbit using a prediction equation as follows: total lipid content (% of live weight, LW)  =  3.33843+0.00248 x LW – 0.00196 x E with LW  =  live weight and E  =  TOBEC measurement.

## Supporting Information

Figure S1 ***In vitro*** **assessment of the efficiency of shRNA expressing constructs.** The OligoWalk web server generated a list of small hairpin RNA candidate sequences ranked by the probability of being efficient to knock down the targeted gene expression. Four sequences (named “a”, “b”, “c”, and “d”) were chosen within this list (their probability of being efficient ranged from 88% to 95.5%), and were tested *in vitro* using a cell transfection assay. These sequences targeted the 3′UTR region of the rabbit *APOBEC1* transcript. The transfections were carried out in CHO.K1 cells (ATCC number CCL-61) using ExGen500 (Euromedex, Souffelweyersheim, France), according to the manufacturer's protocol. The test aimed to measure the efficacy of rbapobec1-shRNA constructs to target the rabbit *APOBEC1* gene expression, in order to select an efficient one that will be used to produce transgenic rabbits. Four constructs harboured the “a”, or “b” or “c” or “d” shRNA sequence. A fifth construct harboured two H1-shRNA genes, one with the “a” and the other with the “d” sequence (see rbapobec1-shRNA diagrams). In the absence of rabbit intestinal cell cultures expressing the *APOBEC1* gene, we designed a chimeric target gene encompassing the *luciferase* gene fused to the targeted sequence of the rabbit *APOBEC1* gene (upper diagram). The 3′UTR of the rabbit *APOBEC1* gene (from nucleotide 756 to 905 respectively to the ATG translation initiation codon) was added at the 3′ position of the *luciferase* gene. Degradation of the 3′UTR region in the target construct by shRNAs was expected to prevent translation of the *luciferase* cistron. Thus, a quantification of shRNA-induced knockdown could be achieved by measuring *luciferase* activity in the transfected cells. The reliability of this method was previously established <Hung, 2006 #30>, showing that it is possible to fuse short target sequences (such as the rabbit *APOBEC1* gene 3′UTR sequences) in the UTR of a reporter gene in order to establish a quantitative reporter-based shRNA validation system. Each shRNA-expressing constructs (0.75 µg/P35 dish) was transfected with the target *luciferase* construct (0.75 µg/P35 dish) or the control empty vector pM10, and the *ß-galactosidase* vector pCH110 (Pharmacia, 1 µg/P35 dish) to correct for transfection efficiency. Luciferase and ß-galactosidase activities were measured 48 h after transfection. The results are given as percentages of luciferse activity in cells transfected by the target *Luciferase* vector and the empty pM10 vector. All luciferase values were normalized to ß-galactosidase activities. The graph is representative of two independent experiments.
(TIF)

Figure S2 **Chromatograms of sequence of DNA amplified from RT-RNA of intestine and liver in wild type or shRNA expressing transgenic animal.** The product of amplification of RT-mRNA encompassing the 2177^th^ codon was sequenced using the APOBR4 oligonucleotide as sequencing primer. By using this oligonucleotide, the antisense strand was sequenced. Three typical chromatograms are reported showing the amplitude of A, C, G and T peaks. The sequence of the sense and the antisense strands are written below. The edited 2177^th^ codon is boxed. The lines and small letters indicate how was measured the height of the peaks. Editing converts the “G” residue of the antisense sequence in a “A” residue”. In the liver in wild type animals (**upper panel**), the APOB mRNA was not edited. A “G” residue was detected at the position of the 2177^th^ codon and no “A” residue was possible to be detected. It was considered that all DNA strands in the amplified sample harbored a CAA codon. In the intestine in wild type animals (**middle panel**), the codon was edited. Most strands harboured a “A” residue in place of the non edited “G” residue. However, a small proportion of strands harboured the “G” residue. The **lower panel** shows that in intestine of transgenic animals expressing the shRNA (sh L21), the height of the “A” peak was reduced and that of the “G” peak was enhanced compared to the chromatogram in intestine of wild type animals. The sample was a mixture of DNA fragments harbouring the CAA or the TAA sequence. The measure of a1, a2, g1 and g2 ensured the determination of the proportion of “G” and “A” containing fragments in the mixture.
(TIF)

Figure S3 **Western blot detection of the human APOBEC1 enzyme in intestinal cell extracts in L02 transgenic rabbits.** Intestinal cell extracts (100 µg of protein in each sample) prepared from a wild type rabbit (WT) and a L02 transgenic rabbit expressing the human APOBEC1 enzyme were fractionated on SDS-PAGE (16%). The human APOBEC1 enzyme was detected by Western blotting using the APOBEC1 antibody (1/1000 dilution). A similar amount of spleen extract was assayed on the same gel as negative control. One specific band (labelled by an arrow) was seen in the L02 transgenic animal at the expected migration rate according to the size of the human protein (27 kD). No band was possible to be detected in the wild type extract or in the spleen extract.
(TIF)

Figure S4 **Plasma triglycerides and cholesterol concentrations in transgenic rabbits expressing the human** ***APOBEC1*** **gene fed with a high fat/high cholesterol diet.** The experiments and symbols are similar to those described in the legend of Figure 8. Rabbits (4 wild type, and 3 transgenic rabbits from line L02) were fed for 8 days with a high fat/high cholesterol diet. Plasma samples were collected before the diet (D0, white bars), after feeding for 8 day with the diet (D8, black bars), after 20 hours starvation (D9 starved, grey bars) and 4 hours after re-feeding with the high fat diet (D9 fed, dotted bars). Triglycerides and cholesterol were assayed as in Figure 7. Values are given in mg/ml, with the standard error of the mean. Comparisons were made between transgenic and wild type animals for each day of the challenge (* = p<0.05).
(TIF)

Figure S5 **Long-term recording of weight curves of rIFABP-hApobec1 transgenic rabbits.** A series of animals (8 wild type; 3 from line L01; 5 from line L02) were weighed for up to 40 weeks after birth. Clearly, the long-term expression of the human APOBEC1 transgene did not induce an excess weigh. Values are means +/− sem.
(TIF)

Table S1 **Sequences of primers.**
(DOC)
