# Supplementary figures and images for: Induction of Body Weight Loss through RNAi-Knockdown of APOBEC1 Gene Expression in Transgenic Rabbits
Source: PLoS One. 2014 Sep 12;9(9):e106655. doi: 10.1371/journal.pone.0106655 (PMC4162549; doi:10.1371/journal.pone.0106655)

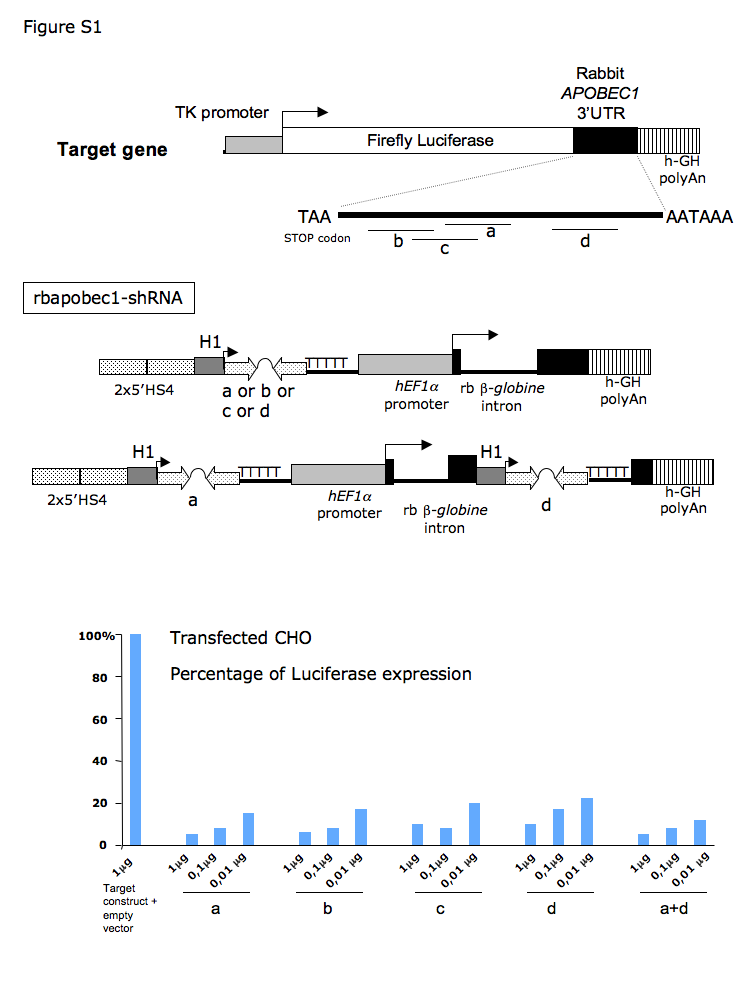

Supplement: Figure S1 — In vitro assessment of the efficiency of shRNA expressing constructs. The OligoWalk web server generated a list of small hairpin RNA candidate sequences ranked by the probability of being efficient to knock down the targeted gene expression. Four sequences (named “a”, “b”, “c”, and “d”) were chosen within this list (their probability of being efficient ranged from 88% to 95.5%), and were tested in vitro using a cell transfection assay. These sequences targeted the 3′UTR region of the rabbit APOBEC1 transcript. The transfections were carried out in CHO.K1 cells (ATCC number CCL-61) using ExGen500 (Euromedex, Souffelweyersheim, France), according to the manufacturer's protocol. The test aimed to measure the efficacy of rbapobec1-shRNA constructs to target the rabbit APOBEC1 gene expression, in order to select an efficient one that will be used to produce transgenic rabbits. Four constructs harboured the “a”, or “b” or “c” or “d” shRNA sequence. A fifth construct harboured two H1-shRNA genes, one with the “a” and the other with the “d” sequence (see rbapobec1-shRNA diagrams). In the absence of rabbit intestinal cell cultures expressing the APOBEC1 gene, we designed a chimeric target gene encompassing the luciferase gene fused to the targeted sequence of the rabbit APOBEC1 gene (upper diagram). The 3′UTR of the rabbit APOBEC1 gene (from nucleotide 756 to 905 respectively to the ATG translation initiation codon) was added at the 3′ position of the luciferase gene. Degradation of the 3′UTR region in the target construct by shRNAs was expected to prevent translation of the luciferase cistron. Thus, a quantification of shRNA-induced knockdown could be achieved by measuring luciferase activity in the transfected cells. The reliability of this method was previously established <Hung, 2006 #30>, showing that it is possible to fuse short target sequences (such as the rabbit APOBEC1 gene 3′UTR sequences) in the UTR of a reporter gene in order to establish a quantitative reporter [file pone.0106655.s001.tif]

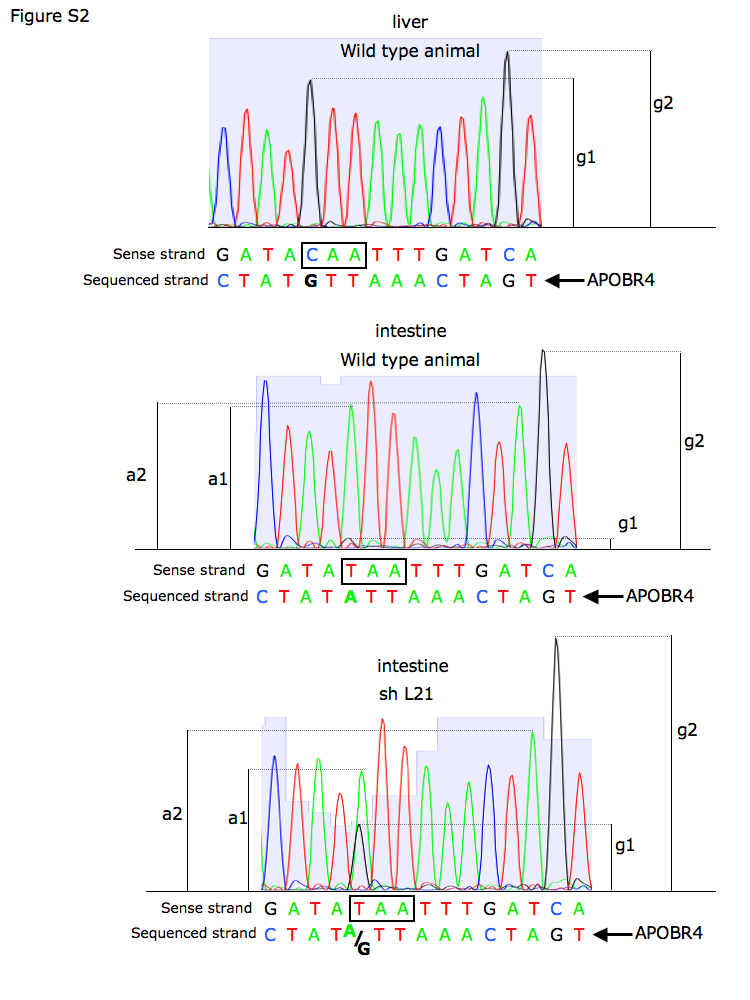

Supplement: Figure S2 — Chromatograms of sequence of DNA amplified from RT-RNA of intestine and liver in wild type or shRNA expressing transgenic animal. The product of amplification of RT-mRNA encompassing the 2177th codon was sequenced using the APOBR4 oligonucleotide as sequencing primer. By using this oligonucleotide, the antisense strand was sequenced. Three typical chromatograms are reported showing the amplitude of A, C, G and T peaks. The sequence of the sense and the antisense strands are written below. The edited 2177th codon is boxed. The lines and small letters indicate how was measured the height of the peaks. Editing converts the “G” residue of the antisense sequence in a “A” residue”. In the liver in wild type animals (upper panel), the APOB mRNA was not edited. A “G” residue was detected at the position of the 2177th codon and no “A” residue was possible to be detected. It was considered that all DNA strands in the amplified sample harbored a CAA codon. In the intestine in wild type animals (middle panel), the codon was edited. Most strands harboured a “A” residue in place of the non edited “G” residue. However, a small proportion of strands harboured the “G” residue. The lower panel shows that in intestine of transgenic animals expressing the shRNA (sh L21), the height of the “A” peak was reduced and that of the “G” peak was enhanced compared to the chromatogram in intestine of wild type animals. The sample was a mixture of DNA fragments harbouring the CAA or the TAA sequence. The measure of a1, a2, g1 and g2 ensured the determination of the proportion of “G” and “A” containing fragments in the mixture. (TIF) [file pone.0106655.s002.tif]

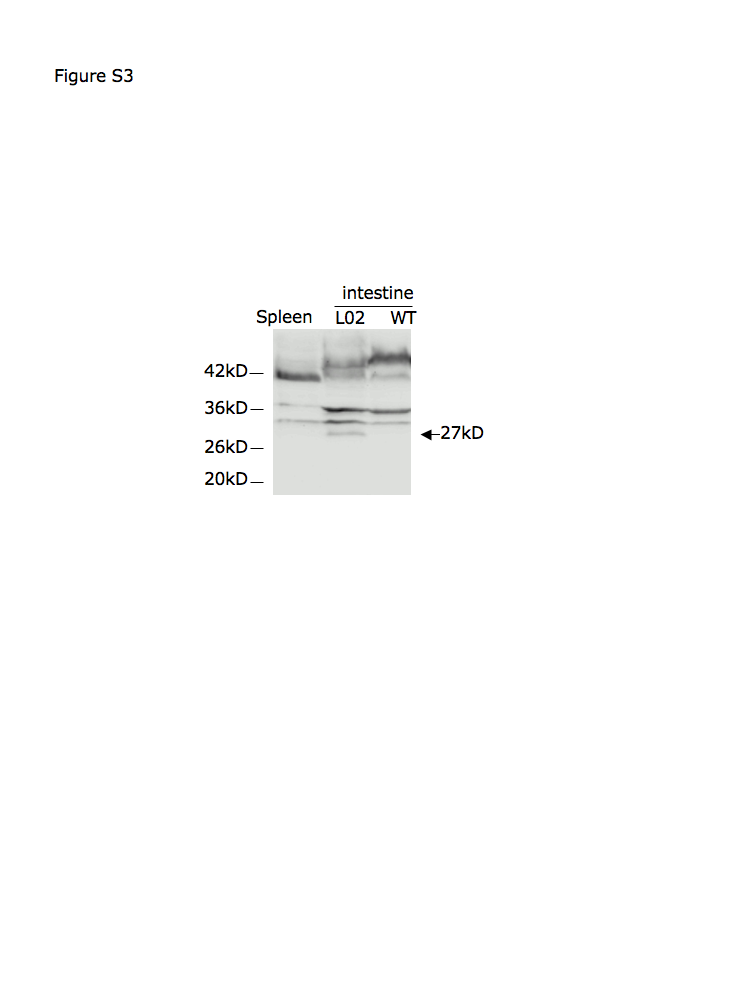

Supplement: Figure S3 — Western blot detection of the human APOBEC1 enzyme in intestinal cell extracts in L02 transgenic rabbits. Intestinal cell extracts (100 µg of protein in each sample) prepared from a wild type rabbit (WT) and a L02 transgenic rabbit expressing the human APOBEC1 enzyme were fractionated on SDS-PAGE (16%). The human APOBEC1 enzyme was detected by Western blotting using the APOBEC1 antibody (1/1000 dilution). A similar amount of spleen extract was assayed on the same gel as negative control. One specific band (labelled by an arrow) was seen in the L02 transgenic animal at the expected migration rate according to the size of the human protein (27 kD). No band was possible to be detected in the wild type extract or in the spleen extract. (TIF) [file pone.0106655.s003.tif]

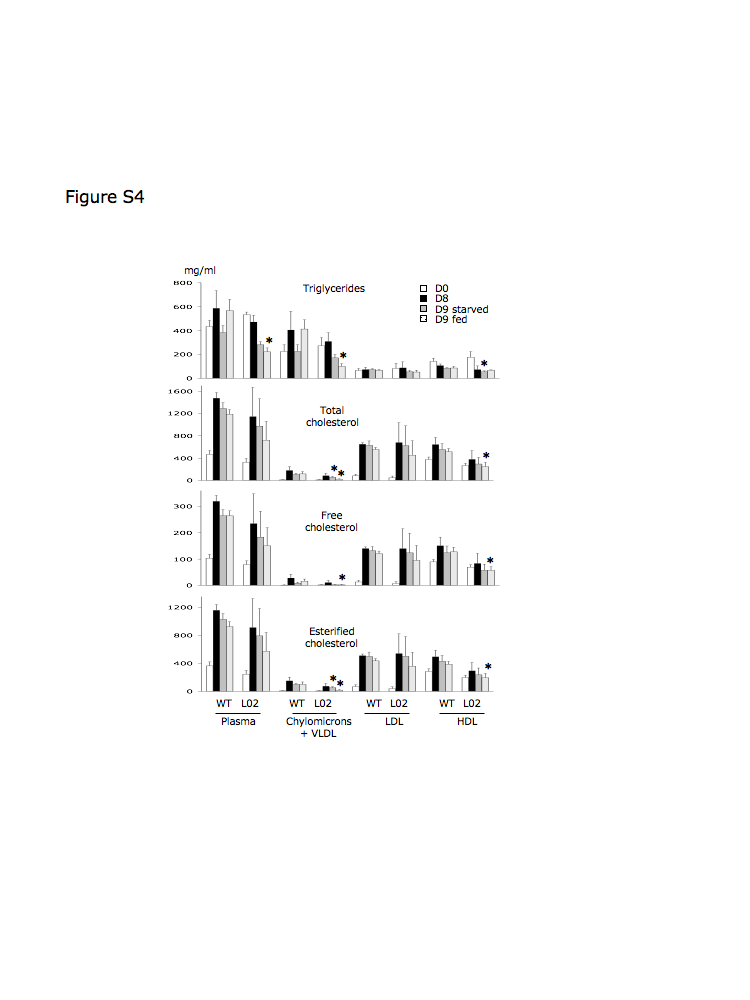

Supplement: Figure S4 — Plasma triglycerides and cholesterol concentrations in transgenic rabbits expressing the human APOBEC1 gene fed with a high fat/high cholesterol diet. The experiments and symbols are similar to those described in the legend of Figure 8. Rabbits (4 wild type, and 3 transgenic rabbits from line L02) were fed for 8 days with a high fat/high cholesterol diet. Plasma samples were collected before the diet (D0, white bars), after feeding for 8 day with the diet (D8, black bars), after 20 hours starvation (D9 starved, grey bars) and 4 hours after re-feeding with the high fat diet (D9 fed, dotted bars). Triglycerides and cholesterol were assayed as in Figure 7. Values are given in mg/ml, with the standard error of the mean. Comparisons were made between transgenic and wild type animals for each day of the challenge (* = p<0.05). (TIF) [file pone.0106655.s004.tif]

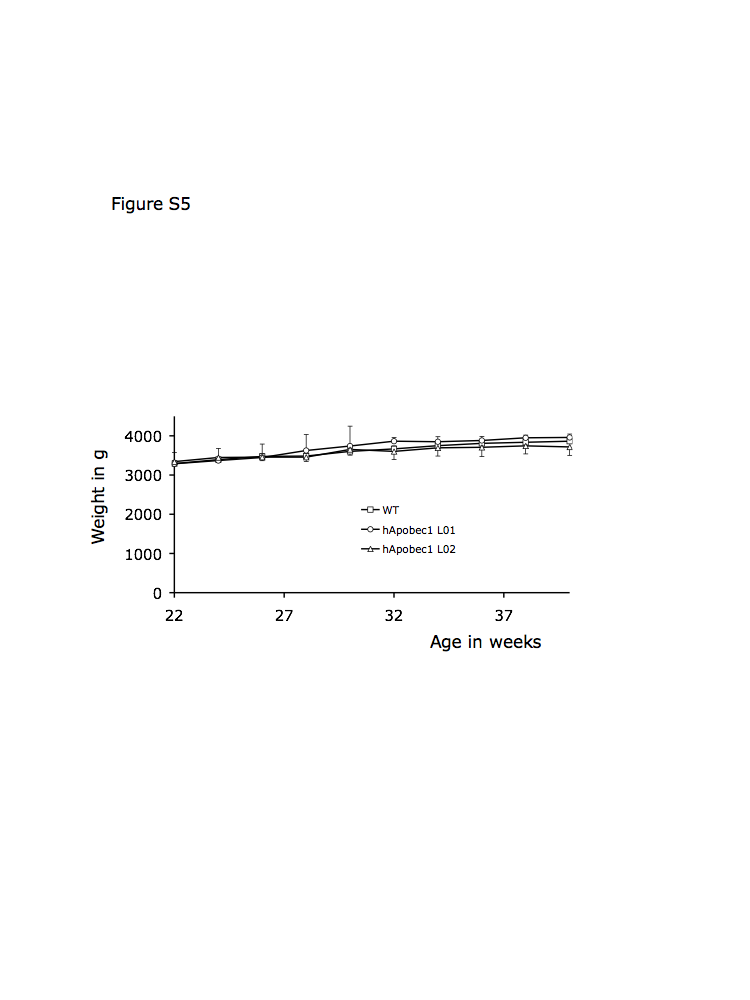

Supplement: Figure S5 — Long-term recording of weight curves of rIFABP-hApobec1 transgenic rabbits. A series of animals (8 wild type; 3 from line L01; 5 from line L02) were weighed for up to 40 weeks after birth. Clearly, the long-term expression of the human APOBEC1 transgene did not induce an excess weigh. Values are means +/− sem. (TIF) [file pone.0106655.s005.tif]
