# Supplementary material for: Induction of Body Weight Loss through RNAi-Knockdown of APOBEC1 Gene Expression in Transgenic Rabbits
Source: PLoS One. 2014 Sep 12;9(9):e106655. doi: 10.1371/journal.pone.0106655 (PMC4162549; doi:10.1371/journal.pone.0106655)
Supplement: Table S1 — Sequences of primers. (DOC) [file pone.0106655.s006.doc]

Table S1 = sequences of primers

|  | position of the primers on the construct | primer name | sequence 5'P-3'OH |
| --- | --- | --- | --- |
| transgene detection and copy number determination | 1 | 5HS4R2 | TGTGCTGGTTTGCAACCCA |
|  |  | 5HS4R2 | GCTGCTCTTTGAGCCTGCAG |
|  | 2 | IFABP F | GAGGACCTGAGTTCAATTCCCA |
|  |  | IFABP R | ACACCACCGCTGTCTTTGGA |
|  | 3 | hapobec F3 | GCTCACTGGCCACAATACCC |
|  |  | hapobec R3 | AGATGATTTTGCCATCTTCTTGAAA |
|  | 4 | hGH F2 | CCACCAGCCTTGTCCTAATAAAA |
|  |  | hGH R2 | GCCCCTTGCTCCATACCA |
|  | 5 | eF1aF5 | CTATGTGGCCAACGCTAAGTGA |
|  |  | eF1aR5 | CCAGGACGGAGTCAGTGAGGAT |
|  | a | 5hs4r1 | AGCACCGCTCTTTGGAGAAG |
|  | b | eF1R104 | TTCCACATGATCTCATGTAGAGGC |
|  | ßcasein gene=copy number calibrator | cas1 | CTCTCATCGCATATTGGAGTGCC |
|  |  | cas2 | GGAACCTGGACTACCATCTGTTGC |
| shRNA assay |  | shRNA (1) | TTAAGAGCACTCTTTGTTGGG |
|  |  | polyT adapter | GCGAGCACAGAATTAATACGACTCACTATAGGTTTTTTTTTTTTVN |
|  |  | universal primer | GCGAGCACAGAATTAATACGACTCACTATA |
|  |  | Let7C | TGAGGTAGTAGGTTGTATGGTT |
| rabbit and human apobec1 gene expression | rabbit apobec1 | lapobec1F | CCAGAGGAAGGAGTCCAGAGTCA |
|  |  | lapobec1R | TCAAATTCCCAGGGTTCAATTC |
|  | human apobec1 (set 3) | hapobec F3 | GCTCACTGGCCACAATACCC |
|  |  | hapobec R3 | AGATGATTTTGCCATCTTCTTGAAA |
|  | reference genes | YWHAZF | GGGTCTGGCCCTTAACTTCTCT |
|  |  | YWHAZR | AGCAATGGCTTCATCAAAAGC |
|  |  | RPL19F | CCAACTCCCGTCAGCAGATC |
|  |  | RPL19R | CAGGGTGTTTTTCCGGCAA |
|  |  | laHPRT F | TTGACACTGGCAAAACAATGCA |
|  |  | laHPRT R | GGGTCCTTTTCACCAGCAGG |
| editing assay |  | APOBR4 | GAATCTGTGTCTTAAACTGTTGCAATATT |
|  |  | LApoB 48F | GGAGACAAGTTTCCAGTGC |
|  |  | LApoB 48R | CAGCCAGGTATGGGATGTTTG |

(1) the sequence of the shRNA was positionned in the 3’UTR of the rabbit *APOBEC1* mRNA. It matched the nucleotides 43 to 64 relative to the STOP codon TAA.
